# Characterization and implications of the dynamics of eosinophils in blood and in the infarcted myocardium after coronary reperfusion

**DOI:** 10.1371/journal.pone.0206344

**Published:** 2018-10-26

**Authors:** Cesar Rios-Navarro, Jose Gavara, Veronica Vidal, Clara Bonanad, Paolo Racugno, Antoni Bayes-Genis, Gema Miñana, Oliver Husser, Ricardo Oltra, Julio Nuñez, Francisco J. Chorro, Vicente Bodi, Amparo Ruiz-Sauri

**Affiliations:** 1 Institute of Health Research-INCLIVA, Valencia, Spain; 2 Cardiology Department, Hospital Clinico Universitario, Valencia, Spain; 3 Medicine Department, School of Medicine, University of Valencia, Valencia, Spain; 4 Cardiology Department and Heart Failure Unit, Hospital Universitari Germans Trias iPujol, Badalona, Spain, Department of Medicine, Universitat Autonoma de Barcelona, Barcelona, Spain; 5 Centro de Investigación Biomédica en Red–Cardiovascular (CIBER-CV), Madrid, Spain; 6 Department of Cardiology, St.-Johannes-Hospital, Dortmund, Germany; 7 Intensive Care Unit, Hospital Clínico Universitario de Valencia, Valencia, Spain; 8 Pathology Department, School of Medicine, University of Valencia, Valencia, Spain; University of PECS Medical School, HUNGARY

## Abstract

**Objective:**

We characterized the dynamics of eosinophils in blood and in the infarcted myocardium in patients and in a swine model of reperfused myocardial infarction (MI). The association of eosinophil dynamics with various outcomes was assessed.

**Methods:**

Serial eosinophil count and pre-discharge cardiac magnetic resonance were carried out in a prospective series of 620 patients with a first ST-elevation MI. In a swine model of reperfused MI, the dynamics of circulating eosinophils and their presence in the infarcted myocardium were determined. In autopsies from chronic MI patients, eosinophils were quantified.

**Results:**

Patient eosinophil count sharply decreased 12h post-reperfusion compared to arrival. A lower minimum eosinophil count was associated with more extensive edema, microvascular obstruction, and infarct size as measured by cardiac magnetic resonance, and also with a higher rate of cardiac events (death, re-infarction, or heart failure) during follow-up. In the experimental model, eosinophil count boosted during ischemia and dropped back immediately post-reperfusion. Myocardial samples revealed progressive eosinophil migration into the infarcted myocardium, especially areas with microvascular obstruction. Markers of eosinophil maturation and survival (interleukin-5), degranulation (eosinophil cationic protein) and migration (eotoxin-1) were detected in the blood of patients, and in porcine myocardium. Eosinophil infiltration was detected in autopsies from chronic MI patients.

**Conclusion:**

Eosinopenia post-MI was associated with an impaired cardiac structure and adverse events. The decay in circulating eosinophils soon after reperfusion mirrors their migration into the infarcted myocardium, as reflected by their presence in heart samples from swine and patients. Further studies are needed to understanding this unexplored pathway and its therapeutic implications.

## Introduction

Eosinophils are granulocytic leukocytes that play an essential role in allergic reactions, asthma, and parasitic infections. Although eosinophils normally account for only 1–3% of circulating leukocytes, the number of both peripheral and tissue eosinophils are markedly altered in some inflammatory reactions [1–4]. Following activation, eosinophils quickly degranulate and release potent factors to promote protective immunity, coagulation, and platelet aggregation [2]. Indeed, they can be also recruited into an inflammatory focus to modulate the immune response [3,4].

In ST-segment elevation myocardial infarction (STEMI), the occurrence of an acute deregulation of the immune system, and its association with the resultant structural myocardial damage and patient outcome has been solidly demonstrated. Indeed, the role of fluctuations in neutrophil and monocyte cell counts and, more recently, in lymphocyte subsets (both in peripheral blood and in the infarcted myocardium) have been widely addressed in this scenario [5–8].

The deleterious effects of eosinophils in other cardiovascular diseases such as eosinophilic myocarditis are well established [4,9]. Preliminary studies in ischemic heart disease have reported an association between eosinophil cell count and a higher event rate and long-term risk of death after myocardial infarction (MI) [10–12]. Furthermore, eosinophils have been histologically detected in intra-coronary thrombi obtained via catheter aspiration in the setting of primary coronary intervention [13,14]. Overall, these observations seem to indicate a potential role of eosinophils in the immune response associated with MI. Nevertheless, a detailed assessment of the dynamics of eosinophils in blood and in the infarcted myocardium after STEMI, as well as the potential structural and clinical implications of these dynamics have been barely addressed so far.

To investigate the association between eosinophils (peripheral and myocardial) and outcomes, we focused on the following specific objectives: 1) To analyse serial eosinophil cell count in peripheral blood of reperfused STEMI patients and determine its association with cardiac magnetic resonance (CMR)-derived edema, microvascular obstruction (MVO), and infarct size as well as with the occurrence of major adverse cardiac events (MACE) during follow-up. 2) To determine serial eosinophil cell count in blood obtained from the coronary sinus and to quantify eosinophil cell infiltration into the infarcted myocardium and in areas with MVO in myocardial samples obtained from a swine model of reperfused MI. 3) To scrutinize the temporal evolution in blood (in patients) and in the infarcted myocardium (in swine) of crucial molecules implicated in eosinophil production, maturation, and recruitment. 4) To quantify the presence of eosinophils in myocardial samples obtained from autopsies of chronic MI patients.

## Material and methods

### Ethics statement

Patient samples were acquired with appropriate ethical permission from the Hospital Clinico Universitario de Valencia´s Ethics Committee. All procedures involving animals were approved by the University of Valencia’s Animal Ethics Committee (2016/VSC/PEA/00074).

### Study in STEMI patients

The study protocol complies with the 1975 Declaration of Helsinki guidelines. All participating patients provided written informed consent.

We prospectively included 691 consecutive patients admitted to a university hospital for a first STEMI between 2004 and 2016 treated with primary coronary intervention and undergoing pre-discharge CMR. Inclusion criteria were stable clinical course during admission, no contraindication to CMR, and no condition related to an alteration of the immune system apart from the MI index.

We excluded 71 patients for the following: death (n = 16), re-infarction (n = 8), severe clinical instability precluding CMR (n = 18), claustrophobia (n = 5), cardiac surgery (n = 3), infections (n = 12), corticoids treatment (n = 7), and leukemia (n = 2). The final study group comprised 620 patients (S1A Fig).

#### CMR

Patients included in the study group were examined with a 1.5 T System (Sonata Magnetom, Siemens, Erlangen, Germany) 7±2 days after STEMI in accordance with our previously validated study protocol [15,16]. CMR studies were analysed offline by an experienced observer blinded to all patient data using customized software (QMASS MR 6.1.5, Medis, Leiden, The Netherlands). Further details on the technical aspects of CMR acquisition, sequences, and quantification can be consulted in the S1 File. Inter- and intra-observer variability for all CMR indices used in the present study is shown in S1 and S2 Tables, respectively.

Left ventricular (LV) ejection fraction (%), LV end-diastolic volume index (ml/m^2^), LV end-systolic volume index (ml/m^2^), and LV mass index (g/m^2^) were determined in cine images. As proxies of the magnitude of structural myocardial damage, the extension of myocardial edema (% of LV mass), infarct size (% of LV mass), and MVO (% of LV mass) were quantified. Myocardial edema was regarded as areas of high T2 signal intensity. All short-axis view slices were separately analysed and the presence of edema was visually quantified by manual planimetry and expressed as percentage of LV mass.

Infarct size (% of LV mass) was assessed as the percentage of LV mass showing late gadolinium enhancement. Microvascular obstruction (MVO,% of LV mass) was quantified by manual planimetry and defined as the percentage of LV mass showing a lack of contrast uptake in the tissue core showing late gadolinium enhancement [15–17].

Based on previously validated data [15–17], the following cut-off values were used to define extensive structural myocardial damage: edema ≥4 segments, MVO >1 segment, and infarct size >30% of LV mass.

#### Follow-Up

MACE consisted of cardiac death, admission for nonfatal re-infarction, or for heart failure, whichever occurred first. Current definitions were applied [18,19]. MACE were systematically reviewed from the medical history of each patient available at hospital database and consensus between 2 cardiologists was required to finally classify a cardiac event.

#### Blood sampling

Total leukocyte counts, and eosinophil counts (x 1,000 cells/ml) were measured upon patient arrival, and at 12, 24, 48, 72, and 96 hours after revascularization by an automated blood cell counter. Patient serum was obtained by centrifuging blood samples at 2500 rpm for 15 min, and immediately stored at -80°C until further analysis (S1B Fig).

#### ELISA

Serial serum samples obtained from STEMI patients were assayed for eosinophil cationic protein (ECP), for interleukin (IL)-5, and for eotaxin-1.

### Study in the experimental swine model

The experimental study conforms to the guide for the care and use of laboratory animals published by the US National Institutes of Health (NIH Publication No. 85–23, revised 1993). Our study protocol has been previously validated [20] and further details can be consulted in the S1 File.

Domestic female pigs (*Sus scrofa*) weighing 25–30 kg were employed. During the surgery protocol, animals were mechanically ventilated employing 50% oxygen gas mixture, and the heart rate, rhythm, and ST-segment changes were continuously monitored.

#### Experimental groups

One control group and five independent MI experimental groups were formed. In the MI groups, after 90-min occlusion of the mid-left anterior descending artery by an angioplasty balloon, experiments were categorized as follows: 1) no-reperfusion, 2) 1-minute, 3) 3-days, 4) 7-days, and 4) 1-month reperfusion (n = 5 each). The control group (n = 5) was subjected to the same experimental protocol used in the MI groups, but without angioplasty balloon inflation, thus ischemia and infarction were not induced.

Further information about macroscopic determination of MVO and infarct size as well as blood sampling is specified in S1 File.

#### Flow cytometry analysis

FITC-CD45 and PE-CD16 conjugated antibodies (Bio-Rad Laboratories, Hercules, CA) were used to quantify eosinophils in whole blood samples obtained throughout the experimental studies. Eosinophils were gated as CD45^+^CD16^-^ cells.

Samples were analysed using a BD FACSVerse flow cytometer (standard 2-laser configuration, BD Bioscience, San Jose, CA), and a minimum of 10,000 events was acquired. FlowJo 8.7 software was applied for the analysis of all the acquired data.

#### Quantitative real-time polymerase chain reaction

The mRNA expression of eosinophil-specific genes (eosinophil peroxidase (EPO), IL-5, and eotaxin-1) was assessed in myocardial samples obtained from the infarcted and remote areas in the six experimental groups.

#### Microscopic study of eosinophil infiltration

Experimental myocardial samples were fixed, embedded in paraffin, sectioned and mounted on glass slides. Hematoxylin-eosin stain (Sigma Aldrich, MI) was performed for histological analysis. Collagen deposition was detected using picrosirius red staining [20]. Luna’s technique was performed for the specific visualization of eosinophil granules [21]. The presence of eosinophils was also assessed by immunohistochemistry using mouse anti-eosinophil major basic protein (EMBP) antibody.

#### Study in autopsies from chronic MI patients

Myocardial samples of three patients with a chronic infarct (more than 6 months after acute MI), and three control subjects were obtained from autopsies.

Presence of eosinophils was histologically evaluated following the same protocol described for the experimental samples (hematoxylin-eosin stain, Luna’s technique, and immunohistochemistry using anti-EMBP antibody).

### Statistical analysis

Data were tested for normal distribution using the Kolmogorov-Smirnov test. Continuous normally distributed data were expressed as the mean ± the standard deviation of the mean and compared using the unpaired Student’s t-test or one-way ANOVA. Non-parametric data were expressed as the median with the interquartile range and compared using the Mann-Whitney U-test or Kruskal-Wallis. Group percentages were compared using the Chi-square test or Fisher’s exact test where appropriate. Linear correlations were assessed using the Pearson’s correlation coefficient. The association of eosinophil minimum count (high>0.04, median value) with time to first MACE was determined by the respective Kaplan-Meier curve and the log-rank test. Statistical significance was considered for two-tailed p-value <0.05. All statistical tests were performed using SPSS 19.0 (SPSS, Inc., Chicago, IL).

## Results

### Dynamics of eosinophil cell count in peripheral blood of STEMI patients. Association with the magnitude of CMR-derived myocardial structural damage and with the occurrence of MACE during follow-up

**Fig 1:**
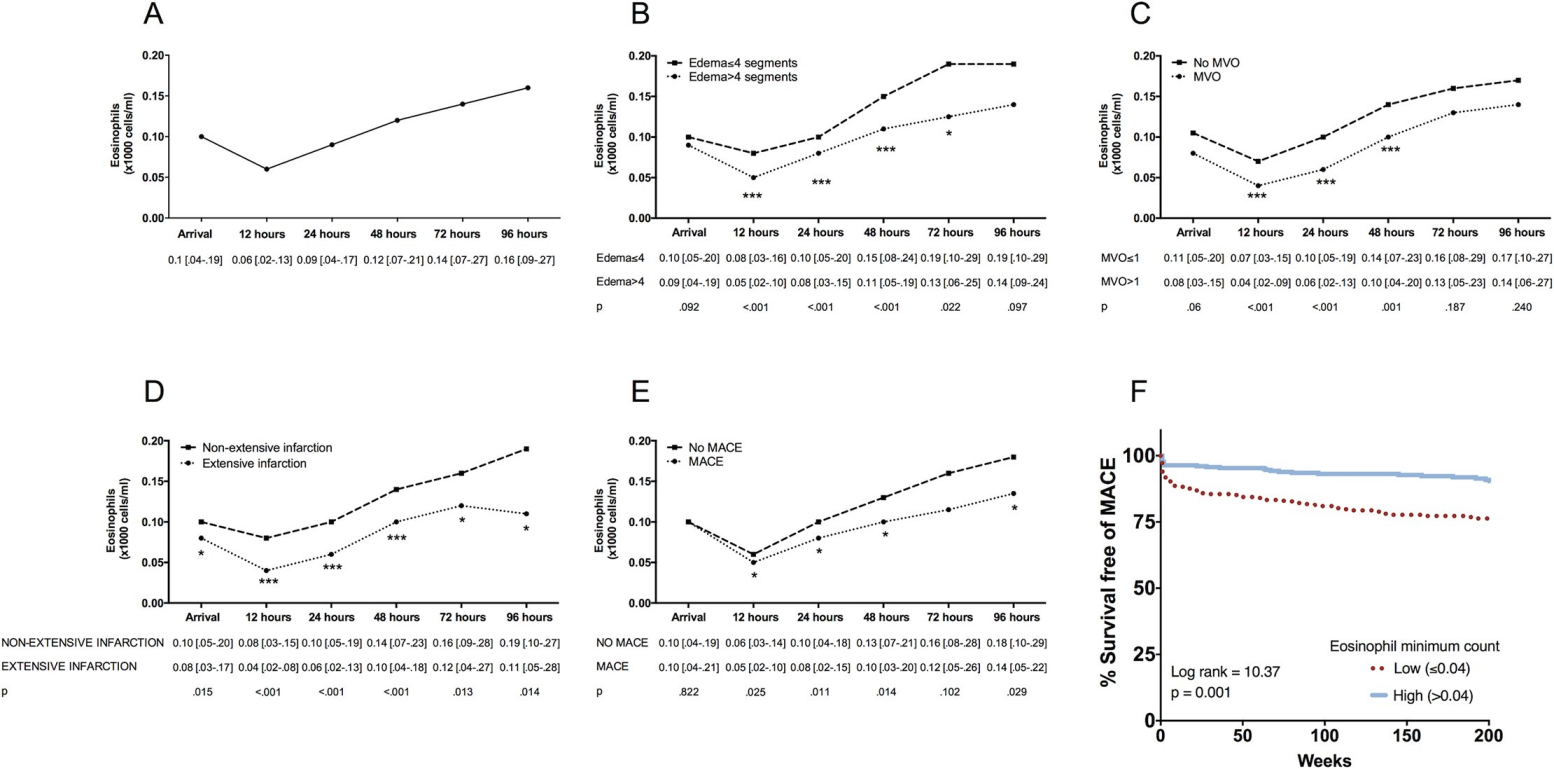
Temporal evolution of eosinophils after reperfused ST-segment elevation myocardial infarction and in relation to edema, microvascular obstruction (MVO), infarct size, and major adverse cardiac events (MACE). Temporal evolution of eosinophils (x1000 cells/ml) (A) in the whole study group and in relation to (B) edema, (C) MVO, (D) infarct size and (E) MACE (cardiac death, re-infarction, or readmission for heart failure) during follow-up. Data are expressed as median with the interquartile range (n = 620) and were analysed by Mann-Whitney U-test. (F) Eosinophil minimum count and outcome Kaplan-Meier curves for survival free of MACE (cardiac death, re-infarction, and readmission for heart failure) during follow-up depending on low eosinophil count. Abbreviations: LV: left ventricular.

The baseline and CMR characteristics of the 620 patients included in the final study are shown in Table 1. The evolution of eosinophil count is displayed in Fig 1A. Characteristically, a sharp drop in eosinophil count occurred in the first measurement (12h) post-revascularization.

**Table 1:** Baseline characteristics, eosinophil counts, and cardiac magnetic resonance (CMR) characteristics of the whole study group.

	Total (*n* = 620)
**Baseline characteristics**	
Age (years)	59±12
Male sex, n (%)	499 (80)
Diabetes mellitus, n (%)	135 (22)
Hypertension, n (%)	301 (49)
Hypercholesterolemia, n (%)	282 (45)
Smoker, n (%)	353 (57)
Heart rate (beats per minute)	78±20
Systolic blood pressure (mmHg)	130±30
Killip class	1.2±0.6
Grace Risk Score	136±32
Timi Risk Score	2 [1–4]
Time to reperfusion (min)	195 [135–300]
Time from chest pain to first medical contact (min)	111 [60–182]
CK-MB mass peak value (ng/ml)	163 [60–290]
ST-segment resolution ≥70%, n (%)	337 (54)
Anterior infarction, n (%)	312 (50)
TIMI flow grade before PCI	1.2±1.3
TIMI flow grade after PCI	2.9±0.5
TIMI flow grade after PCI >3, n (%)	548 (88)
Multivessel disease, n (%)	161 (26)
**White blood cells counts**	
Eosinophils maximum count (x10^3^ cells/ml)	0.2 [0.1–0.3]
Eosinophils minimum count (x10^3^ cells/ml)	0.04 [0.01–0.08]
Leukocyte maximum count (x10^3^ cells/ml)	12.9 [10.6–15.7]
Leukocyte minimum count (x10^3^ cells/ml)	7.9 [6.5–9.6]
Eosinophil to leukocyte ratio maximum (%)	2.4 [1.4–3.6]
Eosinophil to leukocyte ratio minimum (%)	0.3 [0.08–0.8]
**CMR data**	
LVEF,%	52±13
LV end-diastolic volume index (ml/m^2^)	79±23
LV end-systolic volume index (ml/m^2^)	39±20
LV mass (g/m^2^)	73 [63–84]
Infarct size (% of LV mass)	21±15
Edema (% of LV mass)	29±17
MVO (% of LV mass)	0 [0–2.3]

Abbreviations: LV: left ventricle; LVEF: left ventricular ejection fraction; MVO: microvascular obstruction; PCI: primary coronary intervention; TIMI: thrombolysis in myocardial infarction.

#### Association of the dynamics of eosinophil count with the magnitude of the structural myocardial damage

The characteristics of patients with and without CMR-derived extensive edema, MVO, and infarct size are shown in S3–S5 Tables, respectively. The evolution of eosinophil cell count according to the extension of edema, MVO, and infarct size is displayed in Fig 1B–1D, respectively. Patients with extensive edema, MVO, and extensive infarction displayed a significantly lower eosinophil count upon arrival than those without extensive myocardial structural damage. This tendency was sustained throughout all measurements post-revascularization with the nadir being detected at 12h post-reperfusion. Therefore, at all time-points, the lower the eosinophil counts the more severe the myocardial structural damage.

#### Association of the eosinophil count with the occurrence of MACE

During follow-up (median: 90 weeks; range [33–168] weeks), 125 MACE (38 cardiac deaths, 39 nonfatal myocardial infarctions, and 48 readmissions for heart failure) occurred. Baseline characteristics and CMR parameters related to MACE are displayed in S6 Table. The dynamics of eosinophil count within the first hours and days after STEMI in patients with and without MACE during follow-up paralleled that of patients with and without extensive myocardial damage (Fig 1E). Indeed, as shown in the survival curves analysis (Fig 1F), a minimum eosinophil count less than 0.04 x1000cells/ml (median) was strongly associated with a higher probability of MACE. Thus, eosinopenia within the first hours and days after reperfusion of STEMI patients was associated with a higher risk of cardiac events during follow-up.

### Dynamics of eosinophil cell count in coronary sinus blood. Eosinophil cell infiltration into the infarcted myocardium and in areas with MVO in swine

#### Dynamics of eosinophil count in coronary sinus blood in the experimental model

To further characterize the dynamics of circulating eosinophils after MI, especially in the period elapsed between ischemia onset and reperfusion, we investigated the dynamics of circulating eosinophils in a highly-controlled experimental swine model obtaining serial blood samples from the coronary sinus by using flow cytometry. Eosinophils were gated as CD45^+^CD16^-^ cells (S2 Fig).

**Fig 2:**
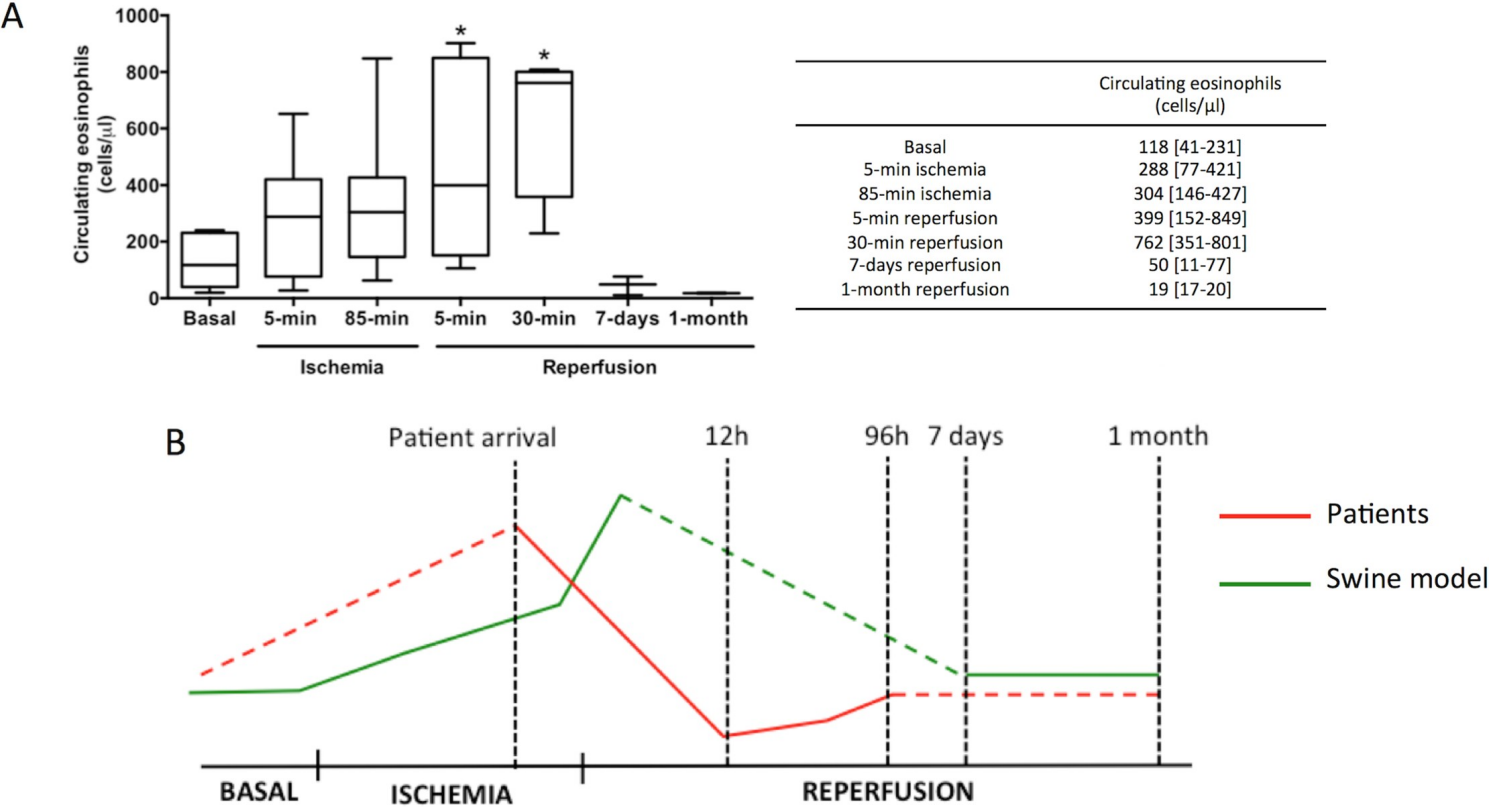
Dynamics of circulating eosinophils in swine subjected to reperfused myocardial infarction (MI). Whole blood was isolated from the experimental model at basal and at different time points of the ischemia and reperfusion process: after 5-min and 85-min of ischemia (immediately before reperfusion) as well as 5-minutes, 30-minutes, 7-days, and 1-month after reperfusion post-MI. Samples were incubated with FITC-CD45 and PE-CD16 and afterwards measured using flow cytometry. (A) Eosinophil counts in whole blood were analysed. Data were expressed as median with the interquartile range (n≥5 independent experiments) and were analysed by Kruskal-Wallis analysis followed by Dunn’s test. *P<0.05 vs. basal. (B) Dynamics of peripheral eosinophils in ST-elevation MI-patients (red) and in the swine model (green). The drop in peripheral eosinophils detected in ST-elevation MI-patients 12h after coronary reperfusion might be preceded by their progressive increase during ischemia and soon after reperfusion, as observed in the experimental model.

Interestingly, a progressive rise in eosinophil cell count took place soon after ischemia onset (coronary occlusion) that reached its maximum value soon (30 min) after reperfusion. Later (7 days and 1 month after reperfusion), eosinophil count dropped to baseline values (Fig 2A).

These results reveal events in a period not accessible for studies in patients and suggest that the fall in circulating eosinophils detected in STEMI patients 12h after coronary reperfusion seems to be preceded by a boost in the number of eosinophils during ischemia and immediately after reperfusion (Fig 2B).

#### Infiltration of eosinophils into the infarcted myocardium

**Fig 3:**
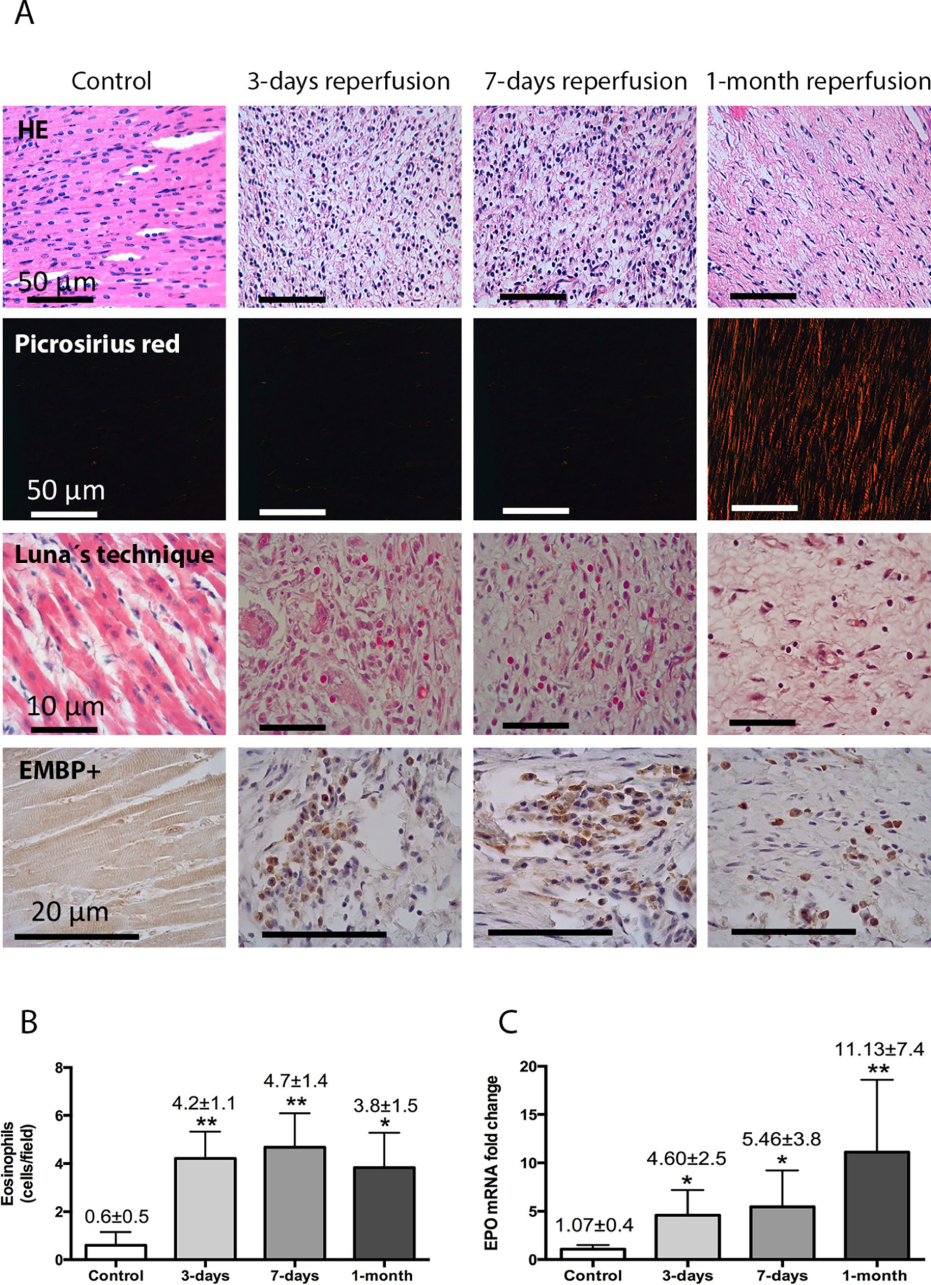
Eosinophil mobilization into the infarcted myocardium in a controlled swine model of reperfused myocardial infarction (MI). (A) Representative images from infarcted tissue isolated from control and three MI groups (90-min of ischemia followed by 3-days, 7-days, and 1-month reperfusion) stained with hematoxylin-eosin (HE) (first panel). The presence of eosinophils was revealed by staining myocardial samples with Luna’s technique, specific for eosinophil granules (second panel) and with the eosinophil-specific protein eosinophil major basic protein (EMBP) (third panel). (B) Quantification of eosinophil cells in the myocardial tissue. Images from the infarcted area isolated from the four independent groups were quantified with Image-Pro Plus analysis software. Scoring was performed by a blinded observer unaware of the experimental group. (C) The expression of eosinophil peroxidase (EPO) in the infarcted myocardium at different times of the ischemia and reperfusion process. Data (mean±SD, n≥4) were analysed by one-way ANOVA analysis followed by Bonferroni test. *P<0.05, **P<0.01 vs. control.

In comparison to controls, histological and morphometric analysis of the infarcted myocardium isolated from the experimental model revealed infiltration of eosinophils at 3 days, 7 days, and 1 month post-reperfusion (Fig 3A and 3B). Eosinophils were not detected in myocardial samples obtained from the non-reperfused group or in those obtained immediately (1 min) post-reperfusion (S3 Fig).

A weak gene expression of EPO (an eosinophil-specific protein) occurred in myocardial samples obtained from controls and from the infarcted area of the non-reperfusion and 1-min reperfusion groups. In contrast, a significant up-regulation of EPO was detected in myocardial samples obtained from the infarcted area of the 3-day, 7-day, and 1-month reperfusion groups (Fig 3C). Neither the histological studies nor the mRNA expression demonstrated an increased eosinophil presence in myocardial samples isolated from the remote areas in any of the five MI groups in comparison with controls (S4 Fig).

In summary, the rapid increase in circulating eosinophils after ischemia onset detected in the experimental model is followed by a marked fall after reperfusion, both in the experimental and clinical models. Migration of eosinophils into the infarcted myocardium might explain the massive loss of circulating eosinophils.

#### Infiltration of eosinophils into the infarcted areas with MVO

To explore the association between the infiltration of eosinophils in the infarcted myocardium following reperfusion and the occurrence of MVO, we carried out 2 studies.

**Fig 4:**
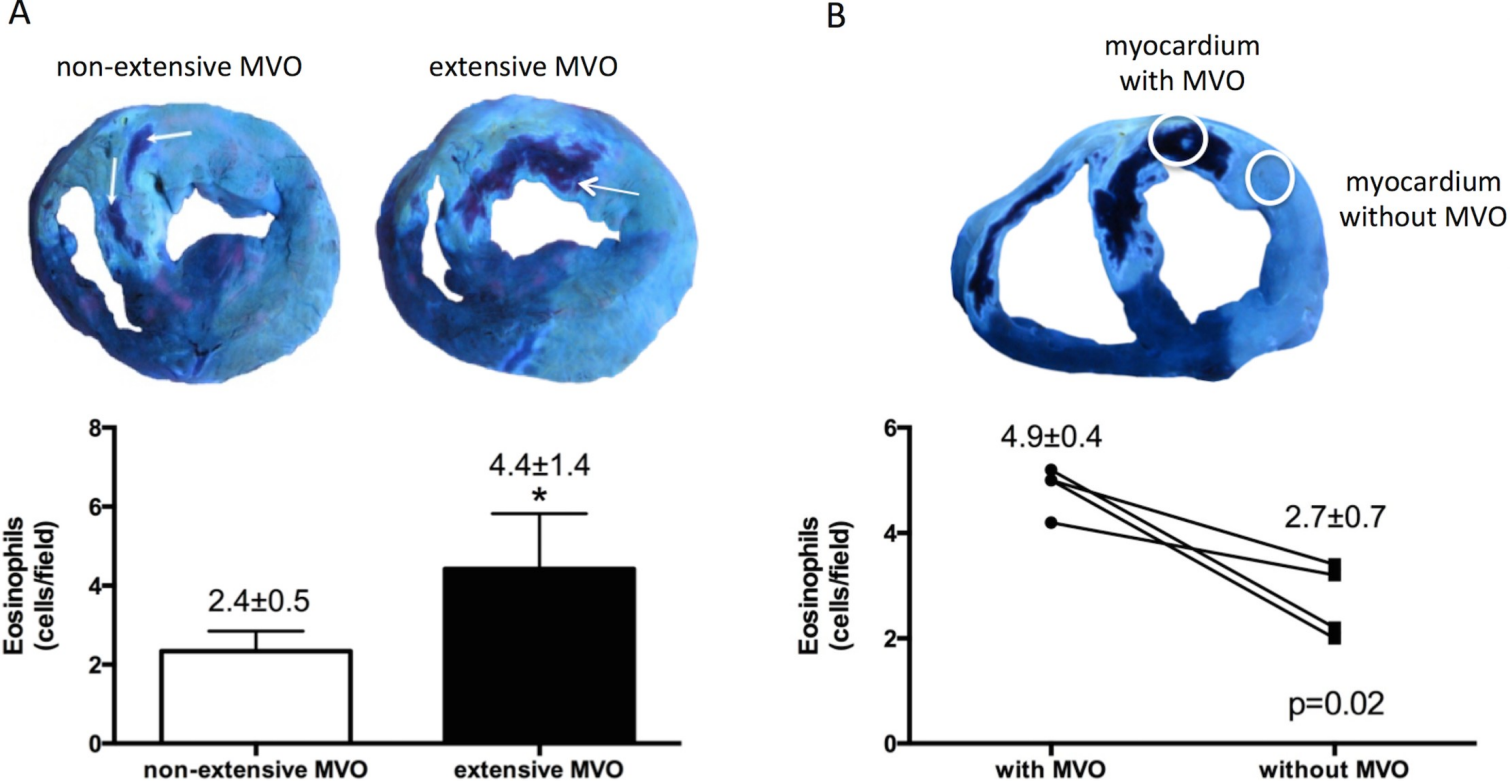
Infiltration of eosinophils into the infarcted areas with microvascular obstruction (MVO). (A) Quantification of infiltrated eosinophils according to the extension of MVO. Animals from the 3-days and 7-days reperfusion groups were categorized according to MVO (extensive: MVO> 5% of area at risk, median value) and the number of eosinophils was morphometrically quantified. (B) Quantification of infiltrated eosinophils in samples obtained from the same heart but comparing infarcted areas with macroscopic MVO and without MVO from the same heart.

Firstly, we analysed the extension of MVO in all experiments with macroscopic evidence of infarction. Two groups were defined: experiments with extensive (MVO> 5% of area at risk, median value), and without extensive MVO. Eosinophil infiltration in the infarct area was significantly higher in experiments classified as extensive MVO (Fig 4A). Representative microscopic images of eosinophil infiltration in hearts with extensive and without extensive MVO are shown in S5A Fig.

Images were quantified with Image-Pro Plus analysis software. Data (mean±SD, n≥4) were analysed by Student’s t test. Scoring was performed by a blinded observer unaware of the experimental group. *P<0.05 vs. non-extensive MVO.

Secondly, in the same experiments used for the previous analysis, each heart was individually evaluated and the magnitude of eosinophilic infiltration in samples obtained from the infarcted areas with macroscopic MVO was morphometrically quantified and compared with those infarcted areas without macroscopic MVO. Of note, eosinophil infiltration was significantly higher in infarcted areas with MVO compared with infarcted areas without MVO (Fig 4B). Representative microscopic images of eosinophil infiltration in areas with MVO and without MVO are shown in S5B Fig.

Therefore, after reperfused MI, eosinophils infiltrate into the infarcted myocardium, and accumulate mainly in areas with MVO. This might in part explain the more extensive CMR-derived myocardial structural damage (especially MVO) and the higher risk of cardiac events detected in STEMI patients with more severe eosinopenia following reperfusion.

### Evolution of crucial cytokines implicated in eosinophil maturation, activation, and recruitment

To investigate the temporal changes in serum levels of IL-5 (an extracellular signal related to eosinophil proliferation, maturation, and survival into the injured tissue), ECP (a cytotoxic molecule secreted after eosinophil activation), and eotaxin-1 (that selectively drives eosinophil recruitment into the damaged area) (S5 Fig), we performed repeated measurements at the pre-defined time-points (upon arrival, and 12h, 96h, and 1-month after reperfusion therapy) in serum obtained from the first 14 STEMI patients included in our final study group and in 10 control subjects. Dynamics of IL-5 and eotaxin-1 genes expression in the infarcted myocardium were investigated in the porcine hearts.

#### IL-5

**Fig 5:**
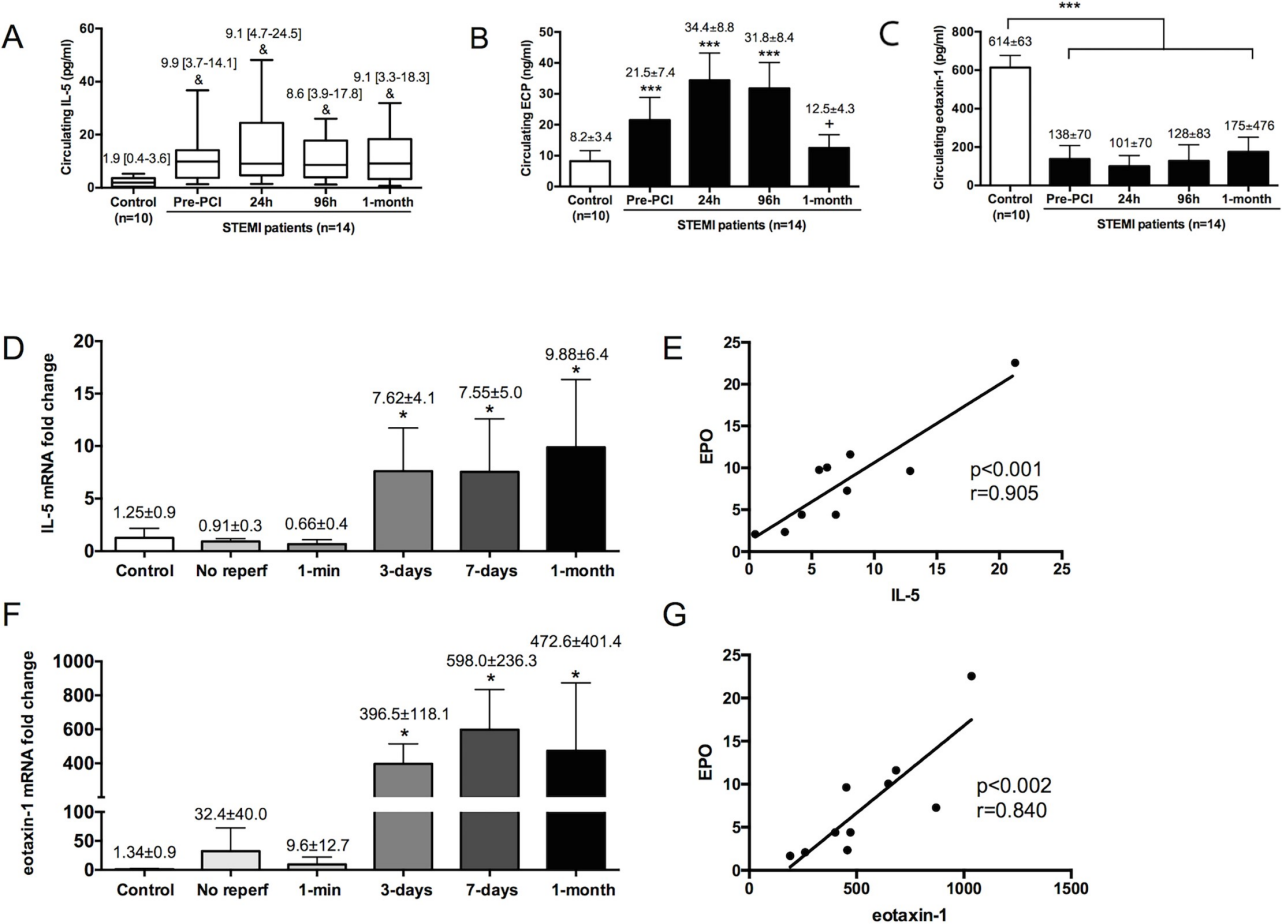
Evolution of crucial cytokines implicated in eosinophil maturation, activation, and recruitment. The serum levels of interleukin (IL)-5 (A), eosinophil cationic protein (ECP) (B), and eotaxin-1 (C) were determined in samples isolated from control subjects (n = 10) and from ST-elevation myocardial infarction (STEMI)-patients (n = 14) upon arrival and after primary coronary intervention (PCI) (24h, 96h, and 1-month). The expression in the infarcted myocardium of IL-5 (D), and eotaxin-1 (F) were obtained at different times of the ischemia and reperfusion process. The correlation of IL-5 and eotoxin-1 with the expression of eosinophil peroxidase (EPO) (E,G, respectively) was assessed using Pearson correlation coefficient. Continuous normally distributed data are expressed as mean±SD and were analysed by one-way ANOVA analysis followed by Bonferroni test. *P<0.05, ***P<0.001 vs. control; ^+^*P*<0.05 vs. 24h post-PCI. Non-parametric data were expressed as the median with the interquartile range and were analysed by Kruskal-Wallis followed by Dunn’s test. ^&^P<0.05 vs. control. Abbreviations: EMBP: eosinophil major basic protein.

In STEMI patients, soluble IL-5 was persistently elevated from arrival (before reperfusion) and throughout the first month after MI (Fig 5A).

In swine, a weak mRNA expression of IL-5 gene occurred in controls and in the infarcted area of the no-reperfusion and 1-min reperfusion groups. Interestingly, a significant up-regulation of IL-5 was detected in myocardial samples obtained from the infarcted area 3 days, 7 days, and 1 month after reperfusion (Fig 5D). Indeed, a positive correlation between mRNA expression of EPO (a specific marker of eosinophil granules and thus of activity) and IL-5 was detected (Fig 5E). These findings suggest that up-regulation of IL-5 occurs first in peripheral blood and then in the infarcted myocardium. This sequence would permit sustained eosinophil production and cell count recovery from onset of ischemia, and eosinophil survival following migration into the infarcted myocardium.

#### ECP

In STEMI patients, circulating ECP was significantly elevated upon arrival (before reperfusion), peaking at 24h and 96h, and returning to values similar to control subjects 1 month after infarction (Fig 5B). These results parallel the rapid boost of eosinophil count detected in porcine blood immediately after coronary occlusion and indicate that in STEMI patients, eosinophil activation and degranulation in blood occurs very early after ischemia onset, even before reperfusion, and returns to control levels 1 month post-reperfusion.

#### Eotaxin-1

Circulating eotaxin-1 levels, a potent eosinophil chemoattractant, were significantly higher in control subjects than in STEMI patients. Indeed, no significant dynamic changes where observed in STEMI patients at different time-points of the ischemia-reperfusion process (Fig 5C).

In porcine hearts, the gene expression of eotaxin-1 was weak in controls and in the infarcted area of the no-reperfusion and 1-minute reperfusion groups. On the contrary, a marked increase in its expression occurred in the 3-day, 7-day, and 1-month reperfusion groups (Fig 5F). Moreover, a positive correlation existed between the mRNA levels of eotaxin-1 and EPO (Fig 5G).

Additionally, the augmented presence of eotaxin-1 in the infarcted tissue was also corroborated by immunohistochemistry (S7 Fig). Eotaxin-1 staining was evidenced in infiltrated leukocytes in the infarcted area of the 3-day and 7-day reperfusion groups, and in interstitial cells of myocardial samples obtained from the infarcted area in chronic phase (1-month reperfusion group). These observations further strengthen the involvement of eotaxin-1 in the recruitment of eosinophils into the infarcted area throughout the sub-acute and chronic phases post-MI.

### Eosinophils in myocardial samples obtained from autopsies of chronic MI patients

**Fig 6:**
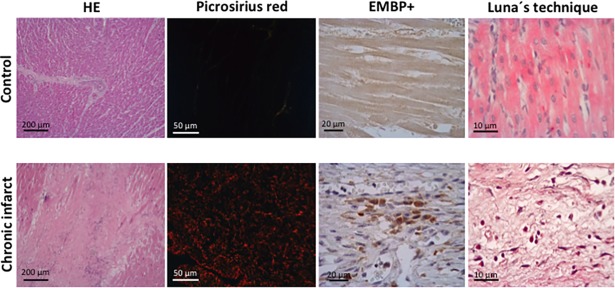
The presence of eosinophils in autopsies from patients with chronic myocardial infarction (MI). Representative images from infarcted tissue isolated from control and chronic MI patients stained with hematoxylin-eosin (HE) (left panel), and picrosirius red (middle-left panel). The presence of eosinophils was revealed by staining myocardial samples with the eosinophil-specific protein eosinophil major basic protein (EMBP) (middle-right panel) and with Luna’s technique, specific for eosinophil granules (right panel).

The presence of eosinophils was also determined in myocardial samples of patients with chronic infarction. Clinical and autopsy characteristics of patients included in the study group are displayed in S7 Table. Similar to the porcine model, histological analyses of the infarcted myocardium revealed that, in comparison with control samples, a significant infiltration of eosinophils occurred in patients with chronic MI (Fig 6). Thus, even in chronic phase, a certain eosinophil infiltration persists in the infarcted area of patients.

## Discussion

We undertook the present study in a large series of STEMI patients and in a highly controlled swine model of MI to gain knowledge on the dynamics, implications, and regulation of eosinophils in the setting of acute reperfused MI (S8 Fig).

### Dynamics of circulating eosinophils in reperfused STEMI

A highly controlled and orchestrated immune response is a necessary step for an adequate repair of the infarcted area. In contrast, an excessive and deregulated activation of the inflammatory cascade has been solidly demonstrated to mediate unnecessary myocardial damage and in turn associates with worse clinical outcomes in STEMI patients [5,6]. Peripheral leukocyte count has been traditionally used as a proxy to explore this association. Neutrophil, monocyte and, more recently, lymphocyte cell counts have been the focus of attention in this scenario [5,7,8]. The pro-coagulant and toxic role of eosinophils in the pathophysiology of other cardiovascular diseases such as eosinophilic cardiomyopathy has been previously demonstrated [9]. Nevertheless, in STEMI patients, eosinophils have been barely explored.

In our study, the association of these dynamics with the resulting myocardial structural damage was evaluated in 620 STEMI patients using the current gold standard non-invasive imaging technique, namely CMR. The occurrence of MACE was monitored during a 90-week median follow-up. Overall eosinophil count dramatically dropped soon after revascularization, observing the more severe the fall, the more extensive the structural myocardial damage in terms of CMR-derived edema, MVO, and infarct size. Unsurprisingly, and in view of the association of post-reperfusion eosinopenia with the magnitude of these potent prognostic parameters, a sharp loss of circulating eosinophils within the first hours after reperfusion was also related to a higher MACE rate in STEMI patients during follow-up.

In line with our results, some studies have reported that a lower eosinophil cell count is associated with a higher risk of cardiac events after MI [10–12]. For instance, Shiyovic and co-workers in a cohort of 2,129 patients showed that an increased peripheral eosinophil cell count after admission for STEMI is associated with a lower risk of death at 1-year follow-up [11]. However, Toor and colleagues concluded that augmented eosinophil count is associated with a lower risk of death at 6 month after reperfusion, and a higher death risk at long-term follow-up [12]. These discrepancies between studies could be due to study methodology, or different cohorts. Our results, obtained in a homogeneous group of STEMI patients treated with percutaneous coronary intervention and with pre-discharged CMR, are in line with those obtained by Shiyovic and co-workers [11] suggesting an association between increased lower circulating eosinophil cell count after admission with high risk of MACE.

Although this finding could be incorporated into our daily armamentarium to improve early risk stratification of STEMI patients, this was not the objective of our research since widely validated scores and recommendations already very well achieve this goal. In our view the ultimate interest of the observations obtained in our clinical series lies in turning attention to the need of a deeper understanding of the true dynamics and regulation (both in peripheral blood and in the infarcted region) of eosinophils from MI onset. For this purpose and in parallel with the clinical research we investigated the course of eosinophils as well as of crucial products and regulators of these cells in a swine model of reperfused MI.

### Dynamics of circulating eosinophils in a controlled experimental model of reperfused MI

The design of clinical studies in MI implies that analyses begin after the first medical contact or more commonly upon patient arrival at the emergency room. This prevents investigation of the dynamics of crucial players in the pathophysiology of MI, in this case inflammatory cells, at the very moment of coronary occlusion and MI onset. For this purpose we used our previously validated porcine model of MI [20] that allowed us to reveal the course of eosinophil count during the period of ischemia (from coronary occlusion until reperfusion). Interestingly a progressive augmentation in peripheral eosinophils was detected during ischemia, peaking immediately after reperfusion (30 min). In parallel to the observations in patients, marked eosinopenia occurred afterwards (Fig 2B). This course suggests a rapid activation and proliferation of theses cells very soon after MI onset, followed by cell loss shortly after reperfusion. This finding would have gone undetected if we had focused our attention only on clinical data and highlights the need for translational research to better understand the complex inflammatory response related to acute MI.

Using the same approach, our group has recently reported that circulating lymphocyte count exhibits a similar pattern as that shown in the case of eosinophils: a rapid proliferation during ischemia and immediately post-reperfusion, followed by a sharp decline afterwards [7]. Research on inflammatory cells in MI has traditionally focussed on neutrophils and monocytes. It has been solidly demonstrated that neutrophil and monocyte counts progressively increase during the first hours and days after coronary reperfusion [5]. Although beyond the scope of the present study, according to these data the course of circulating inflammatory cells subsets seems to display two different patterns following MI: a rapid rise after ischemia onset and a fall after reperfusion in the case of eosinophils and lymphocytes, and a more progressive and sustained increase in the case of neutrophils and monocytes [5,7]. It could be speculated that these patterns may exert an influence on the two waves of myocardial edema following reperfused MI in swine and patients as recently reported [22]. However, further and dedicated research would be needed to verify this and other plausible hypotheses derived from our observations.

### Eosinophil migration into the infarcted myocardium

According to our results, eosinophil cell count boost after MI onset is followed by severe decay in peripheral circulation in the hours after coronary revascularization. The mechanisms underlying this sequence are not yet fully understood. Involvement in coronary thrombi formation [14] might in part explain this vanishing of eosinophils from circulation. In fact, Jiang et al. histologically examined aspirated coronary thrombi after acute coronary syndrome, and all samples displayed eosinophil deposition [13]. However, it is difficult to understand that such a massive loss of peripheral cells could be explained just by eosinophil plugs at the point of occlusion. Moreover in the swine model, where thrombosis is not the leading cause of coronary flow interruption, massive eosinopenia following reperfusion occurred in a similar fashion to that observed in patients. Although accumulation of eosinophils in the occluding thrombus occurs, and may even participate in the initial steps of the pathophysiology of coronary occlusion, the presence of these cells at this point may also be in part the consequence of their retention when making their way towards the infarcted myocardium where release of potent signals massively attract inflammatory cells. Examining our results, it seems progressive migration and infiltration of eosinophils into the infarcted myocardium post-reperfusion of the coronary occlusion are crucial to understanding their rapid decline in the blood stream.

In 1978, Fishbein and coworkers first demonstrated the presence of eosinophils in the infarcted myocardium [23]. Since then, management of MI has dramatically changed and the use of coronary reperfusion therapies is now mandatory. However, as far as we know, no further studies have closely investigated the dynamics of eosinophils cells into the infarcted area. Thus, updating knowledge on this issue under current standards appears highly recommendable.

We histologically detected the presence of eosinophils in the infarcted area soon (3 days, 7 days) and late (1 month) after reperfusion in experimental samples. These cells persisted in myocardial samples obtained from patients late (>6 months) after MI. Migration of eosinophils into injured tissues has been described in many pathological situations, where they undergo degranulation, exert pro-coagulant and toxic effects, and modulate the immune response through different mechanisms [3,4].

Interestingly, eosinophil infiltration was more intense in areas with the most severe structural damage, namely with MVO. This is a multifactorial process characterized by microvascular disruption leading to severe myocardial structural damage. Not surprisingly this phenomenon exerts deleterious effects on LV remodelling and on patient outcomes. The mechanisms underlying microvascular damage in reperfused MI are far from being completely understood [24]. Neutrophil plugging has been traditionally related to the occurrence of MVO. Similar to neutrophils, eosinophils also display potent pro-coagulative and disruptive functions. In fact, eosinophil extracellular traps have been evidenced not only in coronary thrombi but also in a variety of inflammatory pathologies [25]. The presence of eosinophils in zones with MVO in experiments parallels the more severe eosinopenia detected in STEMI patients with MVO. These findings strongly suggest that eosinophils play an important role in the inflammatory response associated with reperfused MI and the resulting structural myocardial damage. Tissue-infiltrating eosinophils are reported to be responsible for modulating acute phase and innate inflammatory reactions. Therefore, a plausible mechanism is that the marked eosinophilia in areas with more severe cardiac damage might provoke a local deregulated inflammatory reaction, and consequently higher myocardial damage [26].

To further clarify the dynamics of eosinophils in this setting, we sequentially determined crucial proteins related to their maturation, activation and recruitment, both in peripheral blood and in myocardial samples.

### Dynamics of crucial molecules related to eosinophils after MI

Eosinophils are granulocytic leukocytes actively involved in numerous inflammatory processes [3,4]. Infiltration into damaged tissue is a complex process orchestrated by a number of crucial cytokines and chemokines (S5 Fig). Briefly, IL-5 is specifically responsible for eosinophil differentiation in bone marrow, activation, and survival both in peripheral blood and in tissue. Eotaxin-1/CCL11, eotaxin-2/CCL24, and eotaxin-3/CCL26 are eosinophil-specific chemokines implicated in eosinophil trafficking and attraction towards the inflammatory focus [1,2]. Although data regarding cardiovascular diseases are scarce, studies related to eosinophilic myocarditis suggest that eotaxin-1 plays a pivotal role in the attraction and migration of eosinophils into the myocardium [4]. Once activated, eosinophils degranulate and release a number of cytokines and chemokines (i.e. EPO, ECP, and EMBP) implicated in coagulation disturbances and tissue disruption among others [1,2]. Although this pathway has been fully described in different pro-inflammatory scenarios, whether the same sequence occurs in the setting of MI is unclear. To investigate this point, we monitored the dynamics of crucial products related to the activity, recruitment, and survival of these cells in peripheral blood and in the infarcted myocardium.

ECP, a marker of eosinophil activity, is released by cell degranulation and participates in the activation of the coagulation cascade. It has been previously reported that ECP can increase in the whole spectrum of stable and unstable ischemic heart disease [27,28]. In STEMI patients we detected raised ECP levels upon patient arrival that returned to control levels 1 month post-reperfusion. This tendency paralleled the rapid boost of eosinophil count detected in porcine blood immediately after coronary occlusion and indicates that eosinophil activation and degranulation in blood occurs very early after ischemia onset, even before reperfusion. It could be speculated that this early activation and degranulation of eosinophils may exert a role in the pro-coagulant milieu in coronary macro- and micro-circulation and as a consequence in the initial stages of edema, MVO, and necrosis.

Eotaxin-1 is a potent eosinophil-specific chemoattractant that has been related, not only to eosinophil recruitment, but also to vascular inflammation [29] and has been implicated in a variety of pathological situations [1,2,4]. A strong up-regulation of this protein was observed in infarcted hearts during acute (3 days and 7 days), and chronic phases after MI. Simultaneously, an enhanced expression of EPO (a marker of eosinophil activity in tissues) and progressive eosinophil infiltration took place. Together, these findings suggest that shortly after the acute degranulation detected in peripheral blood, eotaxin-1 mediates eosinophil trafficking and infiltration, thus enabling the subsequent activity of these cells in the infarcted myocardium.

Finally, involvement of IL-5 has been demonstrated in eosinophil differentiation, activation and survival, both in peripheral blood and in tissue [1,2]. Serum IL-5 levels were persistently elevated in STEMI patients from arrival and throughout the first month of follow-up. In the swine model, IL-5 expression in the infarcted area increased at 3 days and remained elevated at 1 month. These findings corroborate the sequence described for the other products: up-regulation of IL-5 occurs first in peripheral blood and then in the infarcted myocardium. Finally, this sequence permits peripheral cell count recovery and eosinophil survival following migration into the infarcted myocardium.

### Limitations of the study

The present study demonstrates the participation of eosinophils and their products in the pathophysiology of reperfused MI and their association with the magnitude of the myocardial structural damage and the clinical course of patients. The causal role of eosinophils in these associations and the potential therapeutic interventions that could be derived need to be addressed in future studies.

Furthermore, since some differences in eosinophil physiology between humans and swine may exist, further clinical investigation might be carried out in order to continue elucidating the role of eosinophils after MI.

Certain bias towards the exclusion of patients with severe clinical condition might occur.

### Conclusions

In the setting of reperfused MI, eosinophils exhibit important dynamic changes, both in peripheral blood and in the infarcted area. Peripheral eosinophilic cells display a rapid activation immediately after coronary occlusion, followed by a massive loss soon after reperfusion. This sequence induces massive eosinopenia, that in STEMI patients is associated with more severe structural myocardial damage and higher risk of cardiac events. Simultaneously, release of potent mediators induces sustained eosinophil proliferation, activation, and migration into the infarcted area. Finally, the acute peripheral cell count decay appears to be mediated by myocardial infiltration that takes place mainly in zones with the most severe structural damage, namely with MVO, and persists in chronic phases.

The presented results strongly suggest the need for further studies to better understand the pathophysiological role of this almost unexplored pathway as well as the potential therapeutic implications that could be subsequently explored.

## Supporting information

S1 Table Inter-observer variability for traditional cardiac magnetic resonance indices. (DOCX)

S2 Table Intra-observer variability for traditional cardiac magnetic resonance indices. (DOCX)

S3 Table Baseline characteristics, eosinophil counts, and cardiac magnetic resonance (CMR) characteristics of patients with extensive and non-extensive edema. (DOCX)

S4 Table Baseline characteristics, eosinophil counts, and cardiac magnetic resonance (CMR) characteristics of patients with and without microvascular obstruction (MVO). (DOCX)

S5 Table Baseline characteristics, eosinophil counts, and cardiac magnetic resonance (CMR) characteristics of patients with extensive and non-extensive infarction. (DOCX)

S6 Table Baseline characteristics, eosinophil counts, and cardiac magnetic resonance (CMR) characteristics of patients with and without major adverse cardiac events (MACE). (DOCX)

S7 Table Clinical data and autopsy results of patients. (DOCX)

S1 Fig Flow chart of the study group. This flow chart shows the ST-elevation myocardial infarction (STEMI)-patients recruitment (A) and blood sampling (B). MR: magnetic resonance; PCI: primary coronary intervention.
(TIF)

S2 Fig Gating of eosinophils by flow cytometry in swine blood samples. Samples were incubated with FITC-CD45 and PE-CD16 and afterwards measured using flow cytometry. Eosinophils were identified from the rest of leukocytes as CD45+CD16- cells (left panel). Representative histograms from basal (central panel) and 30-min post-reperfusion (right panel) samples were displayed.
(TIFF)

S3 Fig Eosinophil mobilization into the infarcted myocardium in a controlled swine model of reperfused myocardial infarction (MI). (A) Representative images from infarcted tissue isolated from control and two MI groups (90-min of ischemia followed by no reperfusion and 1-min reperfusion) stained with hematoxylin-eosin (HE) (upper panel). The presence of eosinophils was revealed by staining myocardial samples with Luna’s technique, specific for eosinophil granules (lower panel). (B) The expression of eosinophil peroxidase (EPO) in the infarcted myocardium at different times of the ischemia and reperfusion process. Data (mean±SD, n≥4) were analysed by one-way ANOVA analysis followed by Bonferroni test.
(TIF)

S4 Fig Eosinophil mobilization into the remote myocardium in a controlled swine model of reperfused myocardial infarction (MI). (A) Representative images from infarcted tissue isolated from control and five MI groups (90-min of ischemia followed by no reperfusion, 1-min, 3-days, 7-days, and 1-month reperfusion) stained with hematoxylin-eosin (HE) (upper panel). The presence of eosinophils was revealed by staining myocardial samples with Luna’s technique, specific for eosinophil granules (upper panel). (B) The expression of eosinophil peroxidase (EPO) in the remote myocardium at different times of the ischemia and reperfusion process. Data (mean±SD, n≥4) were analysed by one-way ANOVA analysis followed by Bonferroni test.
(TIF)

S5 Fig Representative images of eosinophil infiltration in hearts with (A) extensive and without extensive microvascular obstruction (MVO) and in myocardial regions with (B) MVO and without MVO.
(TIFF)

S6 Fig Pathophysiological mechanism involved in eosinophil trafficking in immune reactions. ECP: eosinophil cationic protein; EMBP: eosinophil major basic protein; EPO: eosinophil peroxidase; IL: interleukin.
(TIFF)

S7 Fig Representative images from infarcted tissue isolated from control and the five myocardial infarction groups stained with eotaxin-1. (TIFF)

S8 Fig Central illustration of the methodology and the main findings of this study. CMR: cardiac magnetic resonance; MACE: major adverse cardiac events; MI: myocardial infarction; MVO: microvascular obstruction; STEMI: ST-segment elevation myocardial infarction.
(TIFF)

S9 Fig Macroscopic representative images of heart slices from an animal subjected to myocardial infarction. Myocardial tissue was stained with thioflavin-S (left panel) and light blue area represents the area at risk. Illustrative images of heart slices stained with 2,3,5-triphenyltetrazolium chloride solution (right panel).
(TIFF)

S1 File Supplementary materials. (DOCX)
