# Supplementary material for: Characterization and implications of the dynamics of eosinophils in blood and in the infarcted myocardium after coronary reperfusion
Source: PLoS One. 2018 Oct 26;13(10):e0206344. doi: 10.1371/journal.pone.0206344 (PMC6203260; doi:10.1371/journal.pone.0206344)
Supplement: S1 Table — (DOCX) [file pone.0206344.s001.docx]

**Supplementary Table 1.** Inter-observer variability for traditional cardiac magnetic resonance indices.

|  | **Relative change** | **Absolute change** | **Coefficient of variation** | **Intra-class correlation coefficient** |
| --- | --- | --- | --- | --- |
| **LVEF (%)** | 4±3% | 2±1% | 0.208 | 0.989 |
| **LV end-diastolic volume index (ml/m²)** | 7±4% | 5±4 ml/m^2^ | 0.301 | 0.981 |
| **LV end-systolic volume index (ml/m²)** | 5±5% | 2±2 ml/m^2^ | 0.508 | 0.994 |
| **LV mass (g/m^2^)** | 8±6% | 6±5 g/m^2^ | 0.249 | 0.944 |
| **Infarct size (% of LV mass)** | 4±5% | 1±1% of LV mass | 0.813 | 0.998 |
| **Edema (% of LV mass)** | 4±6% | 1±2% of LV mass | 0.688 | 0.996 |
| **MVO (% of LV mass)** | 3±4% | 1±2% of LV mass | 1.797 | 0.981 |

**Abbreviations:** LV: left ventricular; LVEF: left ventricular ejection fraction; MVO: microvascular obstruction.
