# Supplementary material for: Characterization and implications of the dynamics of eosinophils in blood and in the infarcted myocardium after coronary reperfusion
Source: PLoS One. 2018 Oct 26;13(10):e0206344. doi: 10.1371/journal.pone.0206344 (PMC6203260; doi:10.1371/journal.pone.0206344)
Supplement: S2 Table — (DOCX) [file pone.0206344.s002.docx]

**Supplementary Table 2.** Intra-observer variability for traditional cardiac magnetic resonance indices.

|  | **Relative change** | **Absolute change** | **Coefficient of variation** | **Intra-class correlation coefficient** |
| --- | --- | --- | --- | --- |
| **LVEF (%)** | 3±2% | 1.5±0.7% | 0.204 | 0.994 |
| **LV end-diastolic volume index (ml/m²)** | 5±3% | 3±2 ml/m^2^ | 0.293 | 0.994 |
| **LV end-systolic volume index (ml/m²)** | 4±5% | 1±2 ml/m^2^ | 0.504 | 0.996 |
| **LV mass (g/m^2^)** | 7±5% | 4±4 g/m^2^ | 0.242 | 0.969 |
| **Infarct size (% of LV mass)** | 3±4% | 0.7±1% of LV mass | 0.807 | 0.998 |
| **Edema (% of LV mass)** | 4±5% | 1±1% of LV mass | 0.685 | 0.998 |
| **MVO (% of LV mass)** | 2±2% | 1±1% of LV mass | 1.908 | 0.993 |

**Abbreviations:** LV: left ventricular; LVEF: left ventricular ejection fraction; MVO: microvascular obstruction.
