# Supplementary material for: Characterization and implications of the dynamics of eosinophils in blood and in the infarcted myocardium after coronary reperfusion
Source: PLoS One. 2018 Oct 26;13(10):e0206344. doi: 10.1371/journal.pone.0206344 (PMC6203260; doi:10.1371/journal.pone.0206344)
Supplement: S3 Table — (DOCX) [file pone.0206344.s003.docx]

**Supplementary Table 3.** Baseline characteristics, eosinophil counts, and cardiac magnetic resonance (CMR) characteristics of patients with extensive and non-extensive edema.

|  | **Non-extensive edema**  **(*n*=325)** | **Extensive edema**  **(*n*=295)** | **p-value** |
| --- | --- | --- | --- |
| **Baseline characteristics** |  |  |  |
| Age (years) | 60±12 | 59±13 | 0.136 |
| Male sex, n (%) | 262 (81) | 237 (80) | 0.931 |
| Diabetes mellitus, n (%) | 74 (23) | 61 (21) | 0.529 |
| Hypertension, n (%) | 170 (52) | 131 (44) | 0.049 |
| Hypercholesterolemia, n (%) | 139 (43) | 143 (49) | 0.155 |
| Smoker, n (%) | 179 (55) | 174 (59) | 0.327 |
| Heart rate (beats per minute) | 76±18 | 81±21 | 0.003 |
| Systolic blood pressure (mmHg) | 135±32 | 125±27 | <0.001 |
| Killip class | 1.2±0.5 | 1.3±0.6 | 0.009 |
| Grace Risk Score | 134±31 | 139±32 | 0.044 |
| Timi Risk Score | 2 [1-4] | 3 [1-5] | <0.001 |
| Time to reperfusion (min) | 210 [142-275] | 251 [150-330] | 0.216 |
| CK-MB mass peak value (ng/ml) | 103 [44-218] | 245 [118-410] | 0.002 |
| ST-segment resolution ≥70%, n (%) | 200 (62) | 137 (46) | <0.001 |
| Anterior infarction, n (%) | 107 (33) | 205 (70) | <0.001 |
| TIMI flow grade before PCI | 1.2±1.4 | 1.2±1.3 | 0.458 |
| TIMI flow grade after PCI | 2.9±0.5 | 2.9±0.4 | 0.523 |
| TIMI flow grade after PCI >3, n (%) | 292 (90) | 256 (87) | 0.470 |
| Multivessel disease, n (%) | 85 (26) | 76 (26) | 0.874 |
| **White blood cells counts** |  |  |  |
| Eosinophils maximum count (x10^3^ cells/ml) | 0.2 [0.2-0.3] | 0.2 [0.1-0.3] | 0.216 |
| Eosinophils minimum count (x10^3^ cells/ml) | 0.05 [0.02-0.1] | 0.03 [0.01-0.06] | <0.001 |
| Leukocyte maximum count (x10^3^ cells/ml) | 12.4 [10.4-14.8] | 13.5 [11.0-16.8] | 0.051 |
| Leukocyte minimum count (x10^3^ cells/ml) | 7.7 [6.5-9.4] | 8.2 [6.5-120.0] | 0.015 |
| Eosinophil to leukocyte ratio maximum (%) | 2.5 [1.6-3.7] | 2.1 [1.2-3.3] | 0.517 |
| Eosinophil to leukocyte ratio minimum (%) | 0.4 [0.1-0.9] | 0.2 [0.06-0.5] | <0.001 |
| **CMR data** |  |  |  |
| LVEF, % | 58±11 | 46±11 | <0.001 |
| LV end-diastolic volume index (ml/m²) | 73±19 | 86±24 | <0.001 |
| LV end-systolic volume index (ml/m²) | 32±15 | 48±22 | <0.001 |
| LV mass (g/m²) | 71 [61-81] | 76 [66-88] | <0.001 |
| Infarct size (% of LV mass) | 13±10 | 29±15 | <0.001 |
| Edema (% of LV mass) | 18±12 | 40±13 | <0.001 |
| MVO (% of LV mass) | 0 [0-0.6] | 1.1 [0-5.4] | <0.001 |

**Abbreviations:** LV: left ventricle; LVEF: left ventricular ejection fraction; MVO: microvascular obstruction; PCI: primary coronary intervention; TIMI: thrombolysis in myocardial infarction.
