# Supplementary material for: Characterization and implications of the dynamics of eosinophils in blood and in the infarcted myocardium after coronary reperfusion
Source: PLoS One. 2018 Oct 26;13(10):e0206344. doi: 10.1371/journal.pone.0206344 (PMC6203260; doi:10.1371/journal.pone.0206344)
Supplement: S4 Table — (DOCX) [file pone.0206344.s004.docx]

**Supplementary Table 4.** Baseline characteristics, eosinophil counts, and cardiac magnetic resonance (CMR) characteristics of patients with and without microvascular obstruction (MVO).

|  | **no MVO**  **(*n*=469)** | **MVO**  **(*n*=151)** | **p-value** |
| --- | --- | --- | --- |
| **Baseline characteristics** |  |  |  |
| Age (years) | 60±12 | 60±13 | 0.118 |
| Male sex, n (%) | 375 (80) | 124 (82) | 0.561 |
| Diabetes mellitus, n (%) | 88 (19) | 47 (31) | 0.001 |
| Hypertension, n (%) | 226 (48) | 75 (50) | 0.752 |
| Hypercholesterolemia, n (%) | 214 (46) | 68 (45) | 0.898 |
| Smoker, n (%) | 263 (56) | 90 (60) | 0.447 |
| Heart rate (beats per minute) | 78±19 | 80±21 | 0.215 |
| Systolic blood pressure (mmHg) | 132±30 | 126±30 | 0.049 |
| Killip class | 1.2±0.5 | 1.3±0.7 | 0.016 |
| Grace Risk Score | 135±31 | 139±34 | 0.350 |
| Timi Risk Score | 2 [1-4] | 3 [1-4] | <0.001 |
| Time to reperfusion (min) | 220 [150-275] | 240 [150-330] | 0.549 |
| CK-MB mass peak value (ng/ml) | 127 [48-257] | 270 [143-429] | 0.001 |
| ST-segment resolution ≥70%, n (%) | 264 (56) | 73 (48) | 0.088 |
| Anterior infarction, n (%) | 204 (44) | 108 (72) | <0.001 |
| TIMI flow grade before PCI | 1.3±1.4 | 0.9±1.3 | 0.002 |
| TIMI flow grade after PCI | 2.9±0.4 | 2.8±0.6 | 0.036 |
| TIMI flow grade after PCI >3, n (%) | 422 (90) | 126 (83) | 0.129 |
| Multivessel disease, n (%) | 114 (24) | 47 (31) | 0.090 |
| **White blood cells counts** |  |  |  |
| Eosinophils maximum count (x10^3^ cells/ml) | 0.2 [0.1-0.3] | 0.2 [0.1-0.3] | 0.050 |
| Eosinophils minimum count (x10^3^ cells/ml) | 0.04 [0.01-0.09] | 0.02 [0.01-0.05] | <0.001 |
| Leukocyte maximum count (x10^3^ cells/ml) | 12.4 [10.4-15.1] | 14.4 [11.7-17.6] | 0.018 |
| Leukocyte minimum count (x10^3^ cells/ml) | 7.7 [6.5-9.5] | 8.5 [6.7-10.1] | 0.003 |
| Eosinophil to leukocyte ratio maximum (%) | 2.4 [1.5-3.6] | 1.9 [1.2-3.5] | 0.029 |
| Eosinophil to leukocyte ratio minimum (%) | 0.3 [0.1-0.8] | 0.2 [0.05-0.4] | <0.001 |
| **CMR data** |  |  |  |
| LVEF, % | 55±12 | 43±11 | <0.001 |
| LV end-diastolic volume index (ml/m²) | 76±22 | 90±22 | <0.001 |
| LV end-systolic volume index (ml/m²) | 35±18 | 52±21 | <0.001 |
| LV mass (g/m²) | 70 [61-80] | 82 [73-95] | <0.001 |
| Infarct size (% of LV mass) | 16±11 | 36±13 | <0.001 |
| Edema (% of LV mass) | 24±15 | 42±14 | <0.001 |
| MVO (% of LV mass) | 0 [0-0.2] | 6.1 [2.8-8.9] | <0.001 |

**Abbreviations:** LV: left ventricle; LVEF: left ventricular ejection fraction; PCI: primary coronary intervention; TIMI: thrombolysis in myocardial infarction.
