# Supplementary material for: Characterization and implications of the dynamics of eosinophils in blood and in the infarcted myocardium after coronary reperfusion
Source: PLoS One. 2018 Oct 26;13(10):e0206344. doi: 10.1371/journal.pone.0206344 (PMC6203260; doi:10.1371/journal.pone.0206344)
Supplement: S5 Table — (DOCX) [file pone.0206344.s005.docx]

**Supplementary Table 5.** Baseline characteristics, eosinophil counts, and cardiac magnetic resonance (CMR) characteristics of patients with extensive and non-extensive infarction.

|  | **Non-extensive infarction**  **(*n*=463)** | **Extensive infarction**  **(*n*=157)** | **p-value** |
| --- | --- | --- | --- |
| **Baseline characteristics** |  |  |  |
| Age (years) | 60±12 | 59±14 | 0.359 |
| Male sex, n (%) | 372 (80) | 127 (81) | 0.881 |
| Diabetes mellitus, n (%) | 90 (19) | 45 (29) | 0.016 |
| Hypertension, n (%) | 228 (49) | 73 (46) | 0.552 |
| Hypercholesterolemia, n (%) | 206 (44) | 76 (48) | 0.395 |
| Smoker, n (%) | 260 (56) | 93 (59) | 0.501 |
| Heart rate (beats per minute) | 76±19 | 86±21 | <0.001 |
| Systolic blood pressure (mmHg) | 131±31 | 127±28 | 0.107 |
| Killip class | 1.14±0.5 | 1.4±0.7 | <0.001 |
| Grace Risk Score | 134±31 | 142±34 | 0.010 |
| Timi Risk Score | 2 [1-4] | 3 [2-5] | <0.001 |
| Time to reperfusion (min) | 186 [130-296] | 210 [150-375] | 0.085 |
| CK-MB mass peak value (ng/ml) | 126 [47-250] | 300 [161-489] | <0.001 |
| ST-segment resolution ≥70%, n (%) | 267 (58) | 70 (45) | 0.009 |
| Anterior infarction, n (%) | 179 (39) | 133 (85) | <0.001 |
| TIMI flow grade before PCI | 1.3±1.4 | 1.1±1.3 | 0.182 |
| TIMI flow grade after PCI | 2.9±0.5 | 2.8±0.5 | 0.339 |
| TIMI flow grade after PCI >3, n (%) | 417 (90) | 131 (83) | 0.019 |
| Multivessel disease, n (%) | 118 (25) | 43 (27) | 0.588 |
| **White blood cells counts** |  |  |  |
| Eosinophils maximum count (x10^3^ cells/ml) | 0.2 [0.1-0.3] | 0.2 [0.1-0.3] | 0.290 |
| Eosinophils minimum count (x10^3^ cells/ml) | 0.04 [0.02-0.09] | 0.02 [0.01-0.04] | <0.001 |
| Leukocyte maximum count (x10^3^ cells/ml) | 12.4 [10.2-14.9] | 14.5 [11.7-18.3] | 0.001 |
| Leukocyte minimum count (x10^3^ cells/ml) | 7.7 [6.5-9.4] | 8.6 [6.9-10.3] | <0.001 |
| Eosinophil to leukocyte ratio maximum (%) | 2.4 [1.6-3.6] | 2.0 [1.1-3.4] | 0.069 |
| Eosinophil to leukocyte ratio minimum (%) | 0.4 [0.1-0.9] | 0.1 [0.04-0.3] | <0.001 |
| **CMR data** |  |  |  |
| LVEF, % | 56±11 | 40±10 | <0.001 |
| LV end-diastolic volume index (ml/m²) | 75±20 | 92±26 | <0.001 |
| LV end-systolic volume index (ml/m²) | 33±15 | 56±23 | <0.001 |
| LV mass (g/m²) | 71 [61-81] | 80 [70-93] | <0.001 |
| Infarct size (% of LV mass) | 14±9 | 41±9 | <0.001 |
| Edema (% of LV mass) | 23±13 | 46±12 | <0.001 |
| MVO (% of LV mass) | 0 [0-0.7] | 4.6 [0.9-8.6] | <0.001 |
|  |  |  |  |

**Abbreviations:** LV; left ventricle; LVEF: left ventricular ejection fraction; MVO: microvascular obstruction; PCI: primary coronary intervention; TIMI: thrombolysis in myocardial infarction.
