# Supplementary material for: Characterization and implications of the dynamics of eosinophils in blood and in the infarcted myocardium after coronary reperfusion
Source: PLoS One. 2018 Oct 26;13(10):e0206344. doi: 10.1371/journal.pone.0206344 (PMC6203260; doi:10.1371/journal.pone.0206344)
Supplement: S6 Table — (DOCX) [file pone.0206344.s006.docx]

**Supplementary Table 6.** Baseline characteristics, eosinophil counts, and cardiac magnetic resonance (CMR) characteristics of patients with and without major adverse cardiac events (MACE).

|  | **NO MACE (*n*=495)** | **MACE (*n*=125)** | **p-value** |
| --- | --- | --- | --- |
| **Baseline characteristics** |  |  |  |
| **Age (years)** | **58±12** | **63±14** | **<0.001** |
| **Male sex, n (%)** | 406 (82) | 93 (74) | 0.055 |
| Diabetes mellitus, n (%) | 109 (22) | 26 (21) | 0.768 |
| Hypertension, n (%) | 234 (47) | 67 (54) | 0.207 |
| Hypercholesterolemia, n (%) | 225 (46) | 57 (46) | 0.977 |
| Smoker, n (%) | 281 (57) | 72 (58) | 0.867 |
| **Heart rate (beats per minute)** | 77±19 | 83±20 | 0.006 |
| Systolic blood pressure (mmHg) | 131±30 | 129±32 | 0.477 |
| **Killip class** | 1.2±0.5 | 1.4±0.7 | 0.003 |
| **Grace Risk Score** | 133±30 | 152±34 | <0.001 |
| **Timi Risk Score** | 2 [1-4] | 3 [1-5] | <0.001 |
| **Time to reperfusion (min)** | 220 [150-275] | 260 [150-350] | 0.015 |
| CK-MB mass peak value (ng/ml) | 154 [57-286] | 182 [76-304] | 0.850 |
| **ST-segment resolution ≥70%, n (%)** | 270 (54) | 67 (54) | 0.071 |
| **Anterior infarction, n (%)** | 237 (48) | 75 (60) | 0.019 |
| TIMI flow grade before PCI | 1.2±1.4 | 1.3±1.4 | 0.386 |
| TIMI flow grade after PCI | 2.9±0.5 | 2.8±0.6 | 0.165 |
| TIMI flow grade after PCI >3, n (%) | 440 (89) | 108 (86) | 0.118 |
| Multivessel disease, n (%) | 121 (24) | 40 (32) | 0.105 |
| **White blood cells counts** |  |  |  |
| Eosinophils maximum count (x10^3^ cells/ml) | 0.2 [0.1-0.3] | 0.2 [0.1-0.3] | 0.467 |
| **Eosinophils minimum count (x10^3^ cells/ml)** | 0.04 [0.01-0.08] | 0.02 [0.01-0.06] | 0.002 |
| Leukocyte maximum count (x10^3^ cells/ml) | 12.7 [10.4-15.2] | 13.5 [11.2-16.6] | 0.256 |
| Leukocyte minimum count (x10^3^ cells/ml) | 7.8 [6.5-9.5] | 8.6 [6.6-10.0] | 0.072 |
| Eosinophil to leukocyte ratio maximum (%) | 2.4 [1.5-3.7] | 2.1 [1.2-3.0] | 0.136 |
| Eosinophil to leukocyte ratio minimum (%) | 0.3 [0.1-0.8] | 0.2 [0.06-0.5] | 0.005 |
| **CMR data** |  |  |  |
| **LVEF, %** | 53±12 | 47±14 | <0.001 |
| **LV end-diastolic volume index (ml/m²)** | 78±22 | 82±26 | 0.091 |
| **LV end-systolic volume index (ml/m²)** | 38±19 | 46±24 | <0.001 |
| **LV mass (g/m²)** | 73 [63-83] | 75 [64-91] | 0.062 |
| **Infarct size (% of LV mass)** | 20±14 | 25±17 | 0.001 |
| **Edema (% of LV mass)** | 28±16 | 33±18 | 0.001 |
| **MVO (% of LV mass)** | 0 [0-2] | 0 [0-2.7] | 0.046 |

**Abbreviations:** LV: left ventricle; LVEF: left ventricular ejection fraction; MVO: microvascular obstruction; PCI: primary coronary intervention; TIMI: thrombolysis in myocardial infarction.
