# Supplementary material for: Characterization and implications of the dynamics of eosinophils in blood and in the infarcted myocardium after coronary reperfusion
Source: PLoS One. 2018 Oct 26;13(10):e0206344. doi: 10.1371/journal.pone.0206344 (PMC6203260; doi:10.1371/journal.pone.0206344)
Supplement: S7 Table — (DOCX) [file pone.0206344.s007.docx]

**Supplementary Table 7.** Clinical data and autopsy results of patients.

|  | **Clinical Data** | **Autopsy Results** |
| --- | --- | --- |
| **Patient 1** | 77-year-old male  Time elapsed since infarction: 7 years  Cause of death: heart failure | Infarct scar area: 2x3 cm lateral wall of the left ventricle  Infarct scar area: 2.5x0.7 cm interventricular septum |
| **Patient 2** | 78-year-old male  Time elapsed since infarction: 1 year  Cause of death: cardiogenic shock | Infarct scar area: interventricular septum |
| **Patient 3** | 55-year-old male  Time elapsed since infarction: 1 year  Cause of death: stroke | Infarct scar area: 2x2 cm interventricular septum |

* In patient 2, infarct size was not quantified, but was visually described in the autopsy report.
