# Supplementary figures and images for: Characterization and implications of the dynamics of eosinophils in blood and in the infarcted myocardium after coronary reperfusion
Source: PLoS One. 2018 Oct 26;13(10):e0206344. doi: 10.1371/journal.pone.0206344 (PMC6203260; doi:10.1371/journal.pone.0206344)

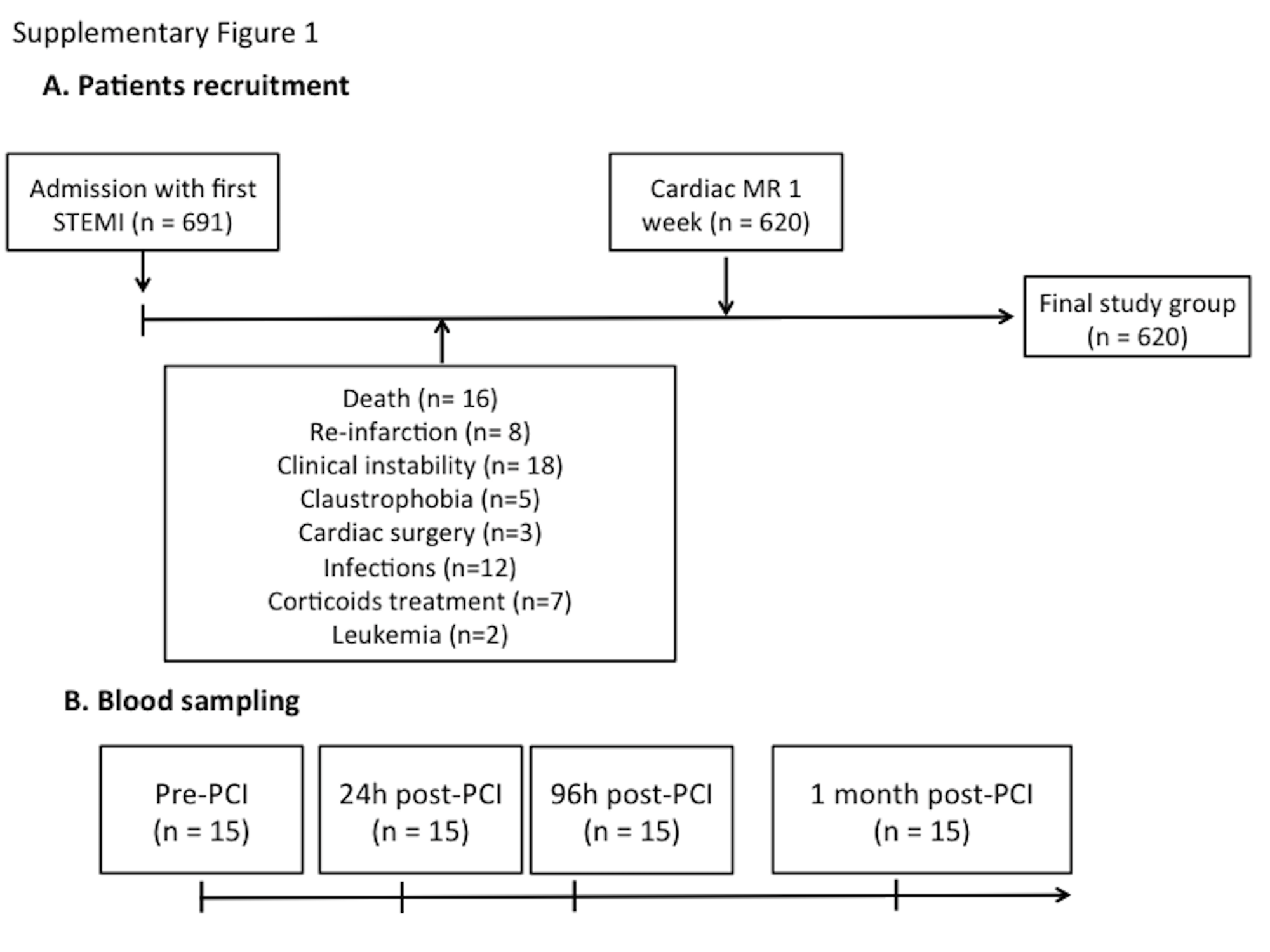

Supplement: S1 Fig — This flow chart shows the ST-elevation myocardial infarction (STEMI)-patients recruitment (A) and blood sampling (B). MR: magnetic resonance; PCI: primary coronary intervention. (TIF) [file pone.0206344.s008.tif]

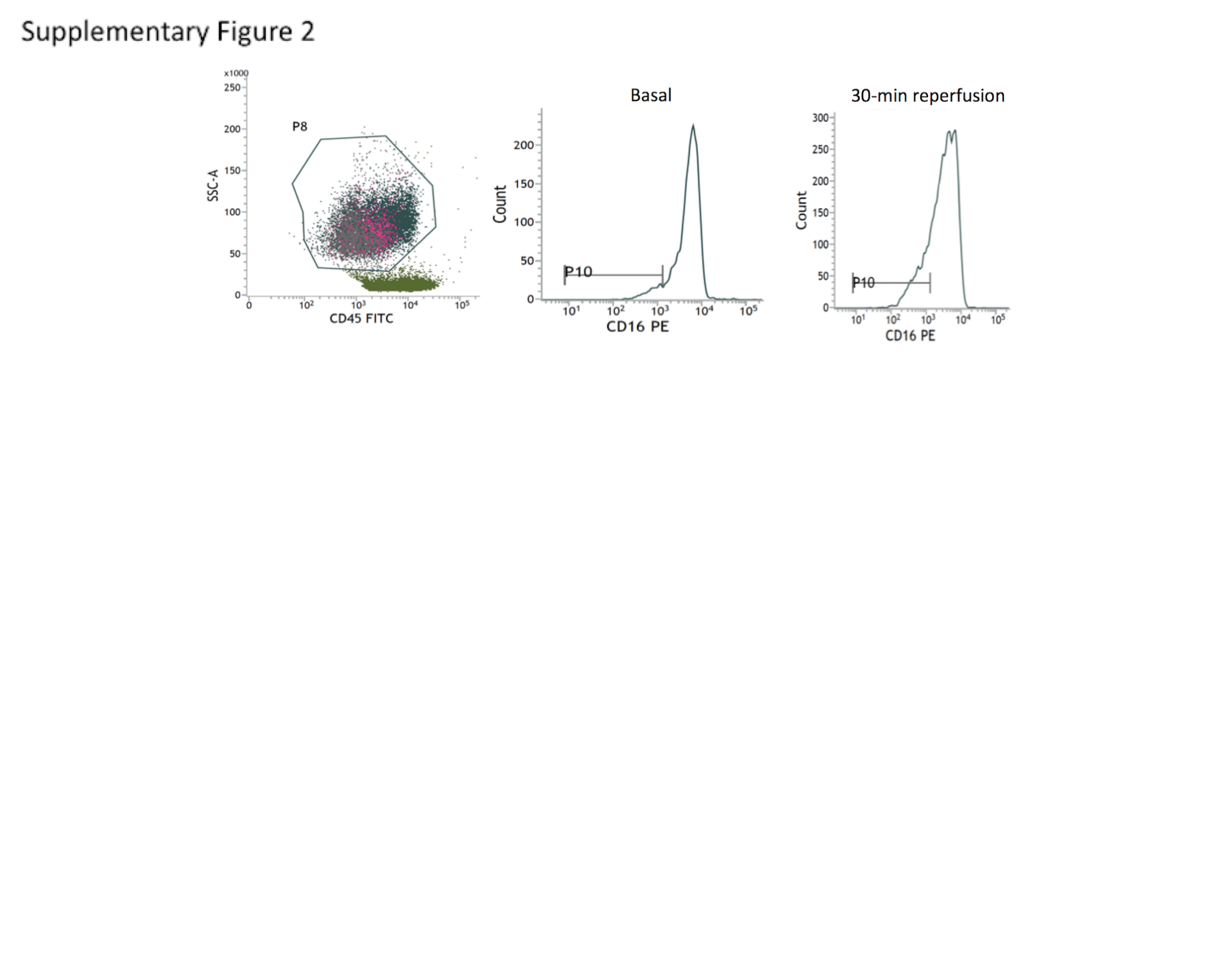

Supplement: S2 Fig — Samples were incubated with FITC-CD45 and PE-CD16 and afterwards measured using flow cytometry. Eosinophils were identified from the rest of leukocytes as CD45+CD16- cells (left panel). Representative histograms from basal (central panel) and 30-min post-reperfusion (right panel) samples were displayed. (TIFF) [file pone.0206344.s009.tiff]

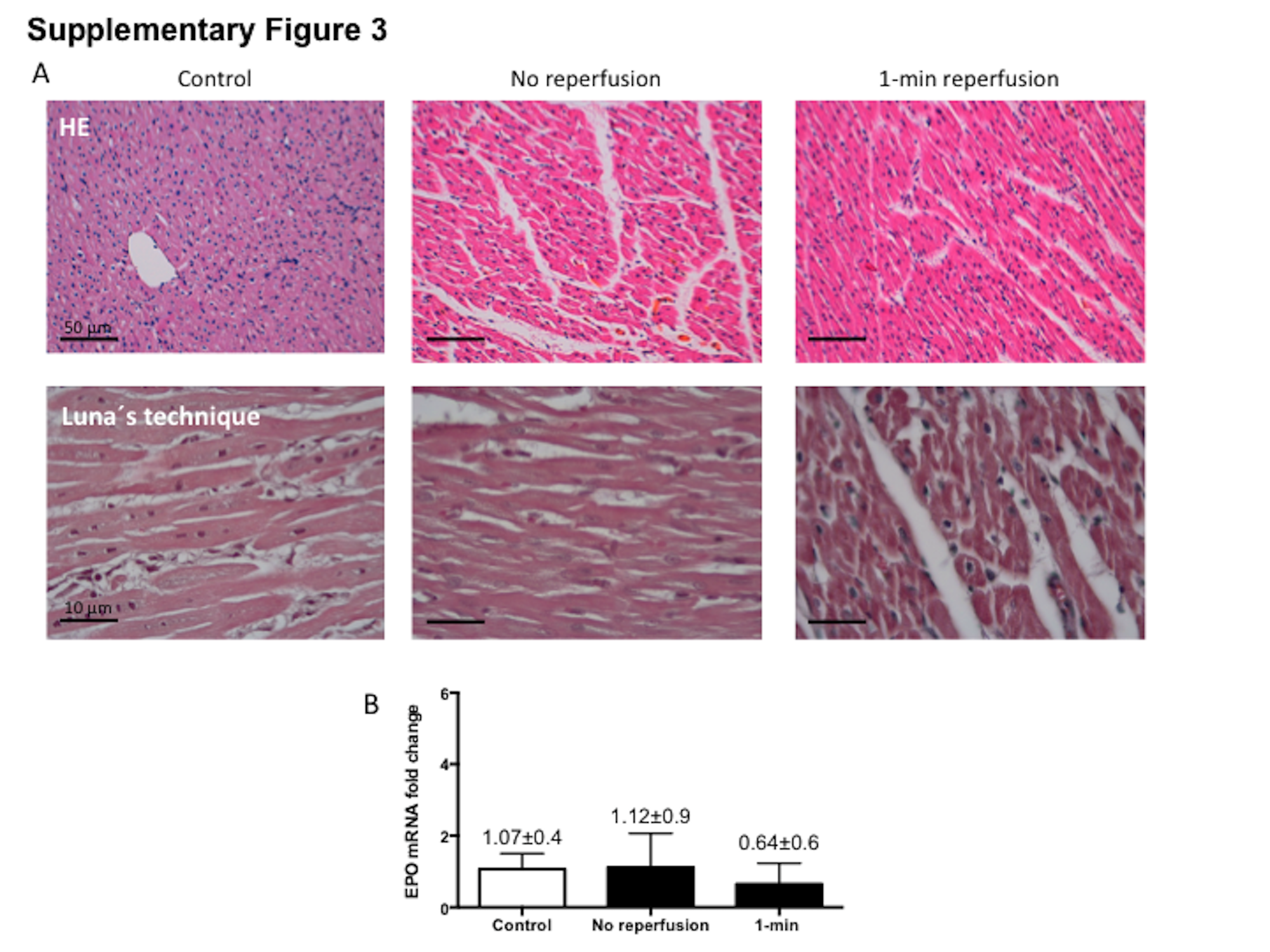

Supplement: S3 Fig — (A) Representative images from infarcted tissue isolated from control and two MI groups (90-min of ischemia followed by no reperfusion and 1-min reperfusion) stained with hematoxylin-eosin (HE) (upper panel). The presence of eosinophils was revealed by staining myocardial samples with Luna’s technique, specific for eosinophil granules (lower panel). (B) The expression of eosinophil peroxidase (EPO) in the infarcted myocardium at different times of the ischemia and reperfusion process. Data (mean±SD, n≥4) were analysed by one-way ANOVA analysis followed by Bonferroni test. (TIF) [file pone.0206344.s010.tif]

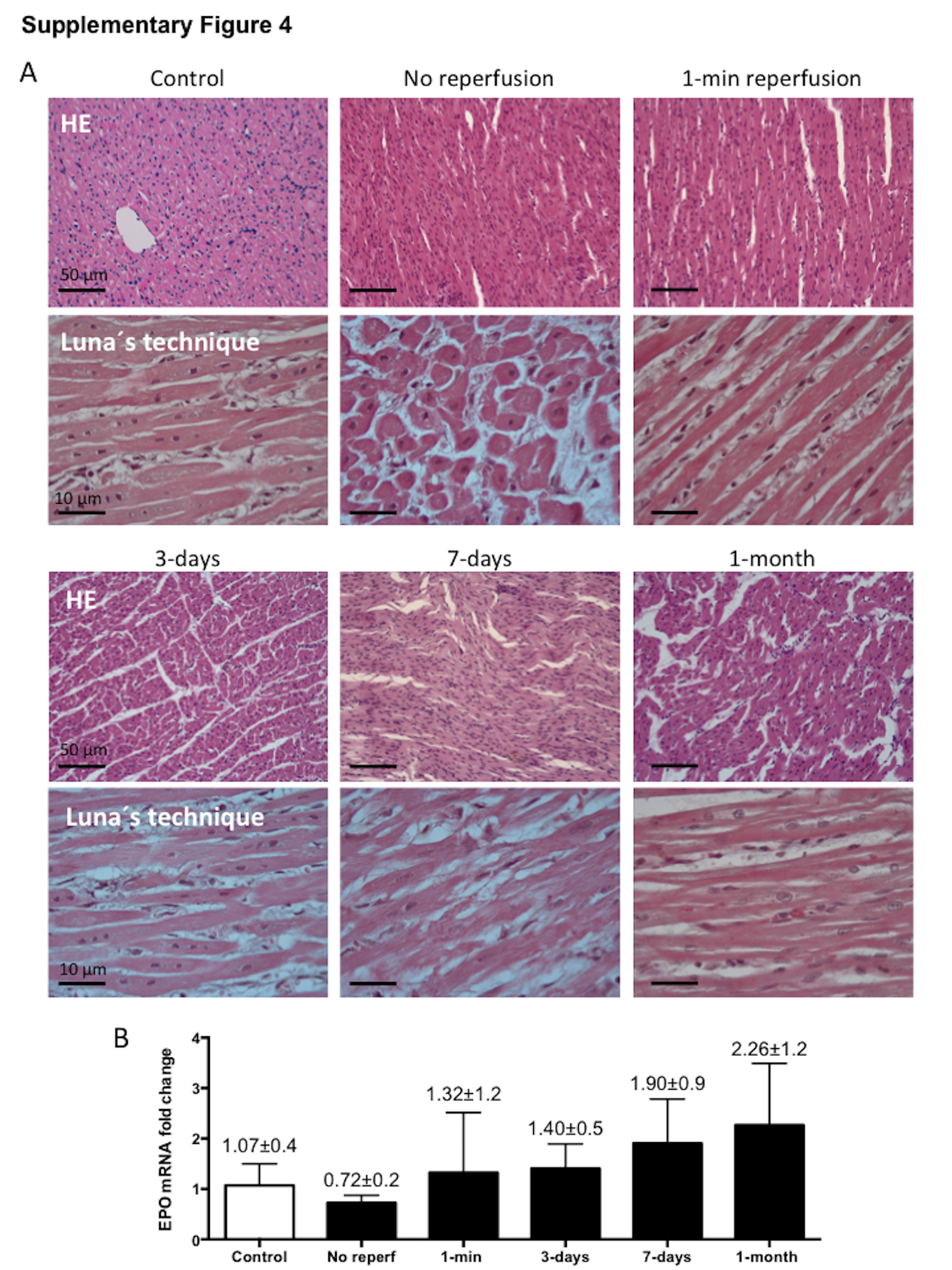

Supplement: S4 Fig — (A) Representative images from infarcted tissue isolated from control and five MI groups (90-min of ischemia followed by no reperfusion, 1-min, 3-days, 7-days, and 1-month reperfusion) stained with hematoxylin-eosin (HE) (upper panel). The presence of eosinophils was revealed by staining myocardial samples with Luna’s technique, specific for eosinophil granules (upper panel). (B) The expression of eosinophil peroxidase (EPO) in the remote myocardium at different times of the ischemia and reperfusion process. Data (mean±SD, n≥4) were analysed by one-way ANOVA analysis followed by Bonferroni test. (TIF) [file pone.0206344.s011.tif]

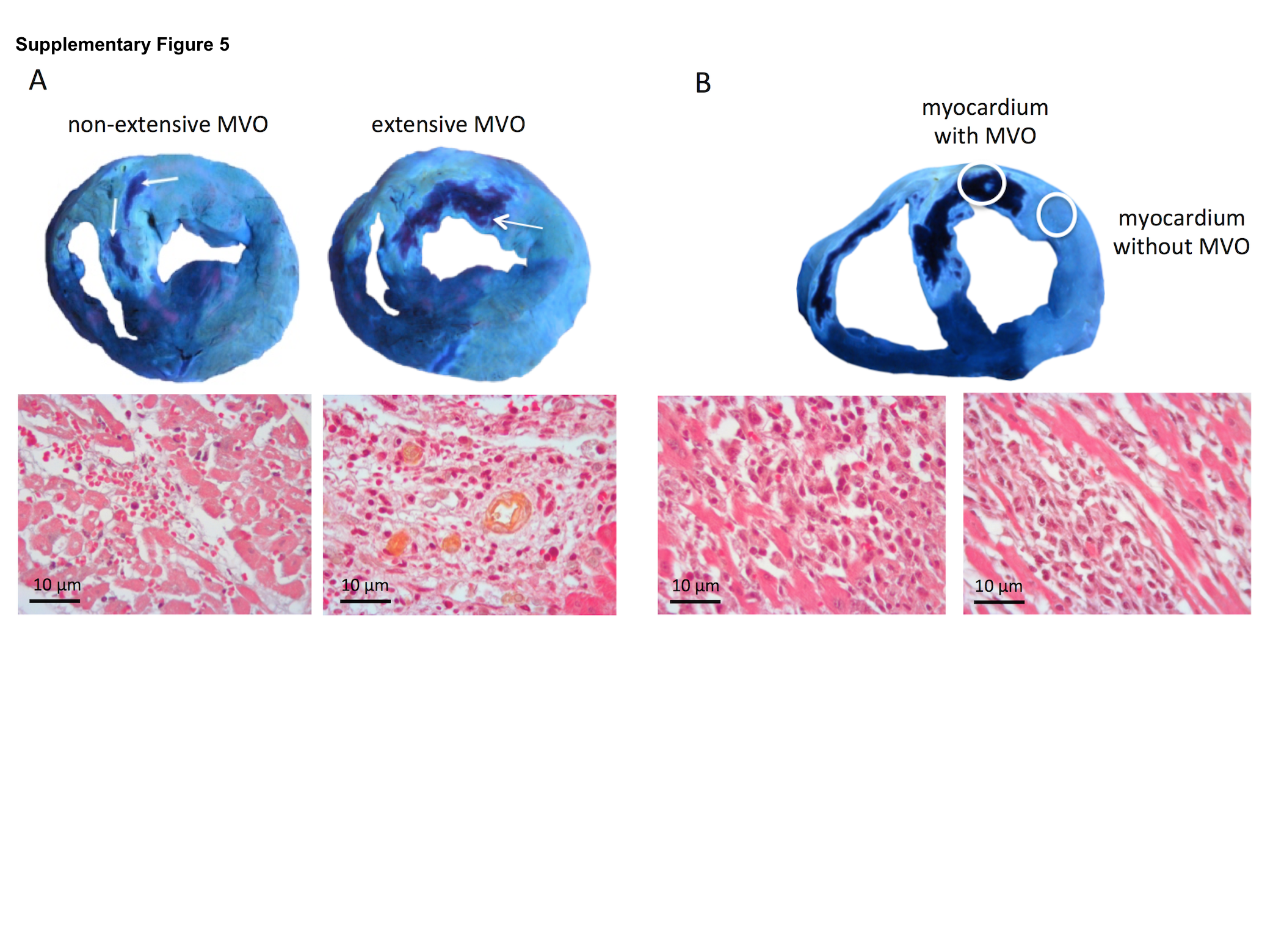

Supplement: S5 Fig — Representative images of eosinophil infiltration in hearts with (A) extensive and without extensive microvascular obstruction (MVO) and in myocardial regions with (B) MVO and without MVO. (TIFF) [file pone.0206344.s012.tiff]

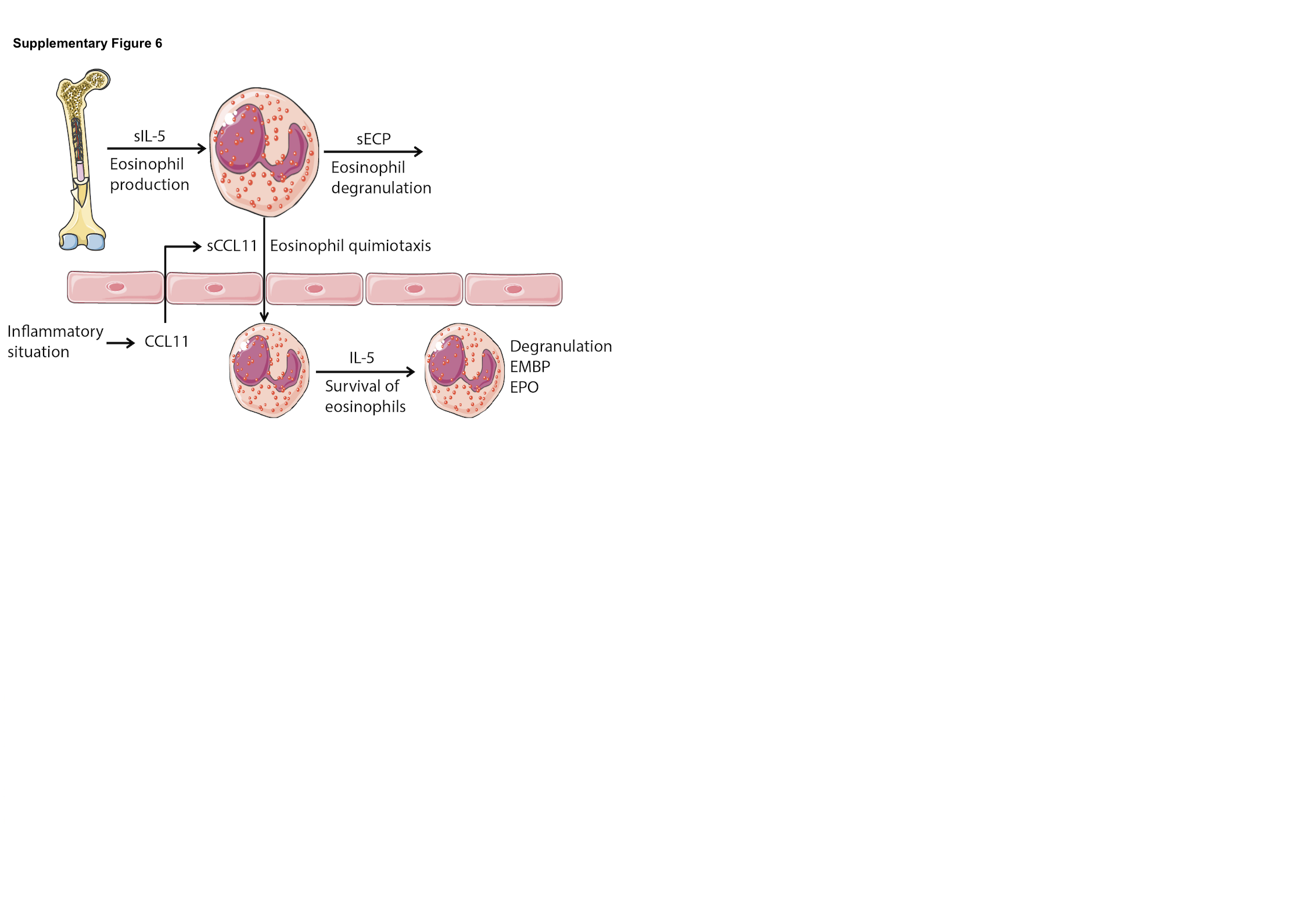

Supplement: S6 Fig — ECP: eosinophil cationic protein; EMBP: eosinophil major basic protein; EPO: eosinophil peroxidase; IL: interleukin. (TIFF) [file pone.0206344.s013.tiff]

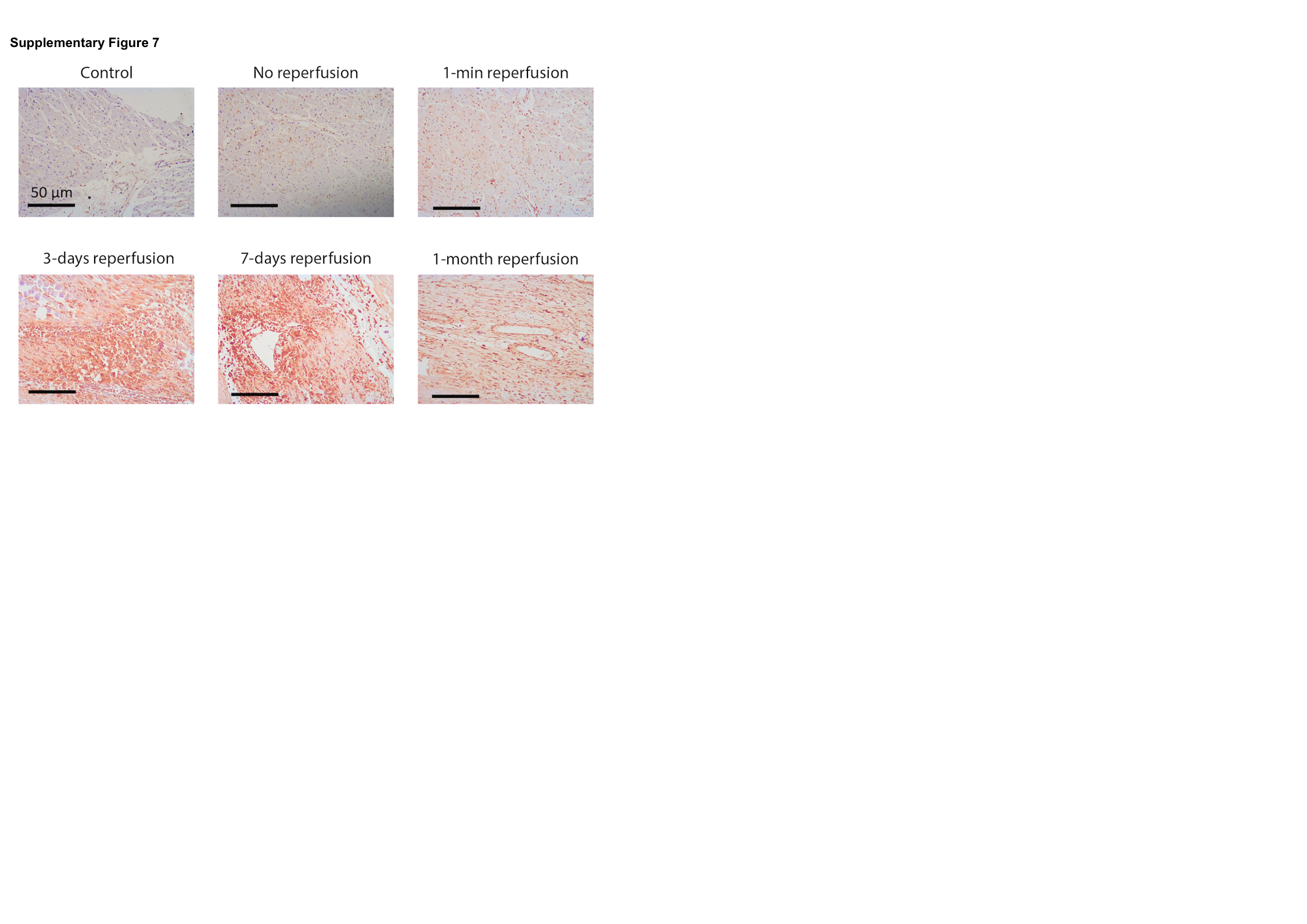

Supplement: S7 Fig — (TIFF) [file pone.0206344.s014.tiff]

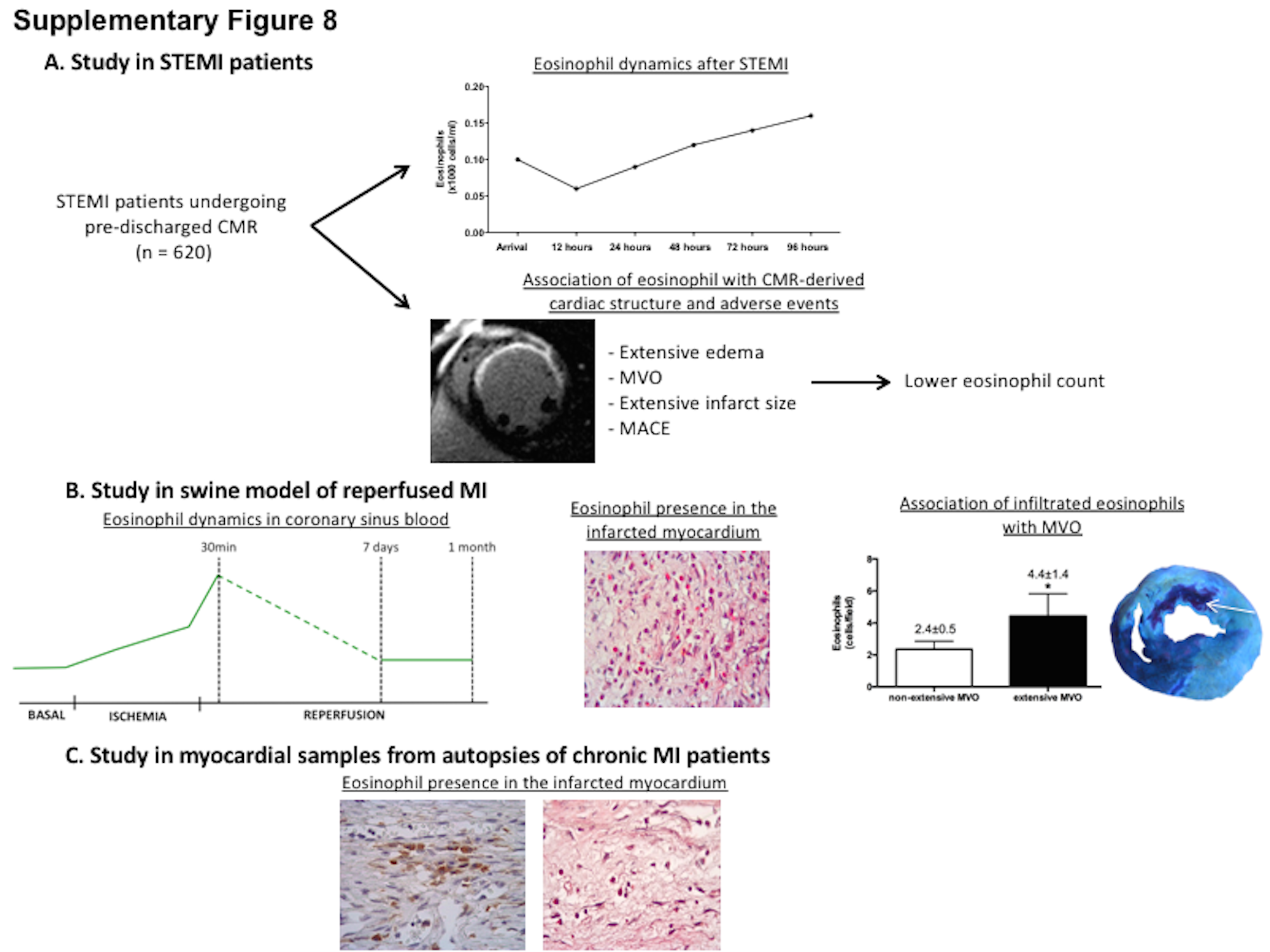

Supplement: S8 Fig — CMR: cardiac magnetic resonance; MACE: major adverse cardiac events; MI: myocardial infarction; MVO: microvascular obstruction; STEMI: ST-segment elevation myocardial infarction. (TIFF) [file pone.0206344.s015.tiff]

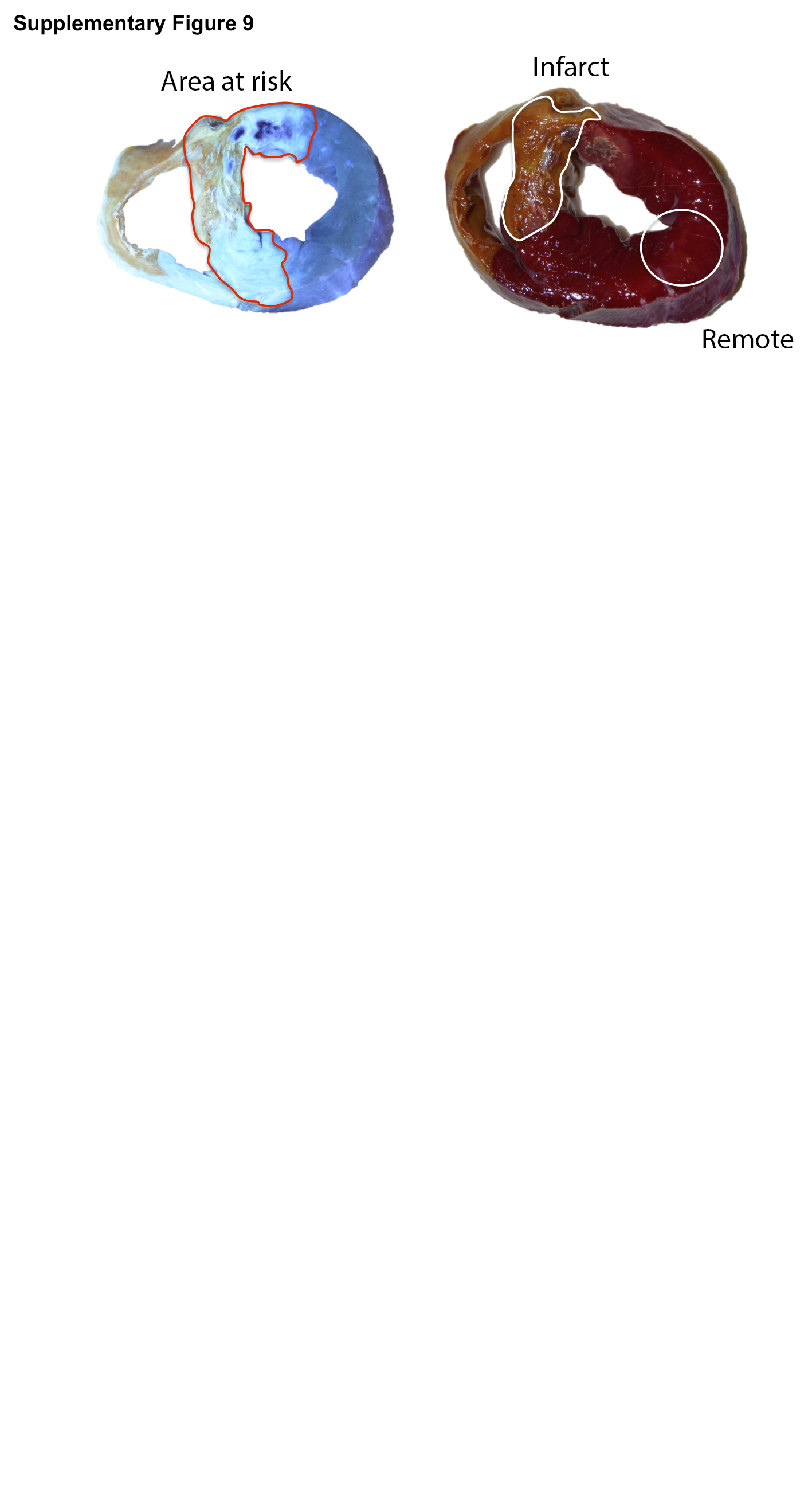

Supplement: S9 Fig — Myocardial tissue was stained with thioflavin-S (left panel) and light blue area represents the area at risk. Illustrative images of heart slices stained with 2,3,5-triphenyltetrazolium chloride solution (right panel). (TIFF) [file pone.0206344.s016.tiff]
