# Supplementary material for: Characterization and implications of the dynamics of eosinophils in blood and in the infarcted myocardium after coronary reperfusion
Source: PLoS One. 2018 Oct 26;13(10):e0206344. doi: 10.1371/journal.pone.0206344 (PMC6203260; doi:10.1371/journal.pone.0206344)
Supplement: S1 File — (DOCX) [file pone.0206344.s017.docx]

**Characterization and implications of the dynamics of eosinophils in blood and in the infarcted myocardium after coronary reperfusion**

**Supplementary material**

**Study in ST-segment elevation myocardial infarction (STEMI)-patients**

**Blood sample and ECG**

Creatine kinase MB mass (ng/ml) was immunologically determined (Immunite assay, Diagnostic Products Corporation, Los Angeles, CA) in at least six consecutive blood samples over the first 72h. The maximum creatine kinase MB mass value was measured.

The percentage of sum of ST-segment resolution 90 min after reperfusion was determined. Complete ST-segment resolution was regarded for a resolution >70%.

**Cardiovascular magnetic resonance acquisition, sequences, and quantification**

Images were acquired by a phased-array body surface coil during breath-holds and were triggered by electrocardiography. All studies were performed by local cardiologists specialized in cardiac magnetic resonance (CMR) with more than 10 years experience and quantified offline by two different operators with 3 years experience blinded to all patient data using customized software (QMASS MR, 6.1.5, Medis, Leiden, The Netherlands). Traditional CMR data were prospectively recorded and immediately included in the registry database [1,2].

Cine images were acquired in two-, three-, and four-chamber views, and in short-axis views using a steady state free precession sequence (repetition time/echo time: 2.8/1.2 ms; flip angle: 58 degrees; matrix: 256 × 300; field of view: 320 × 270 mm; slice thickness: 7 mm) [1,2].

Late gadolinium enhancement imaging was performed 10 to 15 minutes after administering 0.1 mmol/kg of gadolinium diethylenetriaminepentaacetic acid (Magnograf, Juste S.A.Q.F., Madrid, Spain) in the same locations as in the cine images using a segmented inversion recovery steady state free precession sequence (repetition time/echo time: 750/1.26 ms; flip angle: 45 degrees; matrix: 256 × 184; field of view: 340 × 235 mm; slice thickness: 7 mm). Inversion time was adjusted to nullify normal myocardium [1,2].

Black blood, T2-weighted short TI inversion recovery sequences in the same short-axis view as the cine sequences were carried out all in mid-diastole. A half-Fourier acquisition single-shot turbo spin echo multisection sequence was used (recovery time: two R-R intervals; echo time: 33 ms; inversion time: 170 ms; slice thickness: 8 mm; interslice interval: 2 mm; flip angle: 160 degrees; matrix: 256 × 151; bandwidth: 781 Hz/pixel). Additionally, a segmented turbo-spin echo sequence was obtained with one slice per breath-hold (recovery time: two R-R intervals; echo time: 100 ms; inversion time: 170 ms; slice thickness: 8 mm; interslice interval: 2 mm; flip angle: 180 degrees; matrix: 256 × 146; bandwidth: 235 Hz/pixel) [1,2].

Left ventricular (LV) ejection fraction (%), LV end-diastolic volume index (ml/m^2^), LV end-systolic volume index (ml/m^2^), and LV mass index (g/m^2^) were calculated by manual planimetry of endocardial and epicardial borders in short-axis views cine images [1,2].

Areas showing late gadolinium enhancement were visually quantified by manual planimetry. Infarct size (% of LV mass) was assessed as the percentage of LV mass showing late gadolinium enhancement. Microvascular obstruction (MVO, % of LV mass) was quantified by manual planimetry and defined as the percentage of LV mass showing a lack of contrast uptake in the tissue core showing late gadolinium enhancement [1,2].

Myocardial edema was regarded as areas of high T2 signal intensity. All short-axis view slices were separately analysed and the presence of edema was visually quantified by manual planimetry and expressed as percentage of LV mass. Myocardial salvage index was calculated by subtracting the mass of infarcted myocardium from myocardium showing edema and expressed as percentage of LV mass with myocardial edema [1,2].

**ELISA**

Serial serum samples obtained from STEMI patients and control subjects were assayed for eosinophil cationic protein using the human ECP ELISA Kit (Aviscera Bioscience INC, Santa Clara, CA), for interleukin-5 using human IL-5 Quantikine ELISA Kit (R&D Systems, Minneapolis, MN), and for eotaxin-1 using human eotaxin-1 Quantikine ELISA Kit (R&D Systems, Minneapolis, MN) following manufacturer´s instructions.

**Study in swine model**

**Reperfused myocardial infarction model in swine**

Intramuscular ketamine (8 mg/kg) and medetomidine (0.1 mg/kg) for sedation and continuous intravenous 10-mg/kg/h infusion of 2% propofol for anaesthesia induction were administrated. Indeed, in order to diminish life-threatening arrhythmias, pigs were pre-treated with intravenous amiodarone (300 mg) and lidocaine (30 mg). A 7 Fr sheath was introduced into the right femoral artery for monitoring blood pressure and to access the left anterior descending coronary artery (LAD). A 7 Fr Amplatz Left 0.75 catheter was used to selectively engage the proximal LAD and a standard hydrophilic angioplasty wire was advanced and placed in the distal LAD. A 2.5 mm x 15 mm over-the-wire angioplasty balloon was inflated at 6 atm in the mid LAD distal to the first diagonal branch. Coronary artery occlusion was confirmed by contrast injection and by electrocardiographic ST-segment elevation [3,4].

**Macroscopic study: area at risk, MVO, and infarct size quantification**

In all cases, immediately before sacrifice, 20 mL of 4% thioflavin-S solution was infused into the LAD through the lumen of an over-the-wire balloon that was positioned and inflated at the same point used for the induction of MI. Thereafter, the heart was arrested with potassium chloride and excised.

Once the heart was excised, the left ventricle was sectioned into 5-mm thick short-axis slices. Firstly, to determine the area at risk and the area with MVO in the territory perfused by the LAD artery, each slide was viewed under ultraviolet light and photographed. Secondly, to determine the infarcted area, slices were incubated into 2% 2,3,5-triphenyltetrazolium chloride solution at 37ºC for 20 min, viewed under room light and photographed (S9 Fig) [3,4].

Light blue represents the thioflavin-S myocardial uptake after infusion through the LAD, while dark blue indicates a lack of LAD-perfusion. As a consequence, MVO was defined as dark blue zones in the core of the LAD-perfused area and was expressed as the percentage of the LAD-perfused area.

The infarct area was defined as the triphenyltetrazolium chloride-negative myocardial region, and was expressed as the percentage of the LV volume.

After digitalizing the images, manual offline quantification of all short-axis slices was performed in a dedicated laboratory (Cardiac Imaging Unit, INCLIVA, Valencia, Spain) by a trained observer unaware of the experimental protocol applied. All numerical data analysis was performed in the software package MATLAB 8.4. The predefined slice thickness (5 mm) and the presence of a ruler beside heart slices in all images were required to calculate LV myocardial volumes [3,4].

**Blood sampling**

Blood samples at baseline (before coronary occlusion), during ischemia (5 min and 85 min after balloon inflation) and after reperfusion (5 min, 30 min, 7 days, and 1 month after balloon deflation) were obtained from a multipurpose catheter placed in the coronary sinus.

**Quantitative real-time Polymerase Chain Reaction**

In order to extract RNA, RNeasy Plus Mini Kit (QIAGEN GmbH, Hilden, Germany) was employed following the manufacturer’s instructions. Gene expression was determined by real time Polymerase Chain Reaction using a 7900HT Fast Real-Time Polymerase Chain Reaction System (Applied Biosystems, Thermo Fisher Scientific, Waltham, MA). The values of the threshold cycle (Ct) were calculated and normalized to the housekeeping gene GAPDH.

We used specific primers pre-designed by Bio-Rad Laboratories (Hercules, CA) for analysis of porcine eosinophil peroxidase (qSscCED0018488), interleukin-5 (qSscCID00005451), eotaxin-1 (qSscCED0017221), and the endogenous GAPDH (qSscCED0017494).

**Immunohistochemistry**

For immunohistochemistry, after peroxidase inactivation (H2O2 0.3%) and blockade with horse serum, sections were incubated overnight (4ºC) with the following primary antibodies diluted in PBS/BSA 0.1%: anti-human eosinophil major basic protein (dilution 1:10, cat# sc-59164, Santa Cruz Biotechnology, Santa Cruz, CA), and rabbit anti-swine eotaxin-1 (dilution 1:150, cat# KP1374S, Kingfisher Biotech, St Paul, MN). Specific labelling was detected with a biotin-conjugated goat anti-mouse or goat anti-rabbit secondary antibody (1:500 dilution, Dako Glostrup, Denmark).

**Morphometric quantification of eosinophil presence in myocardium**

Photographs at 63x magnification were taken in five independent fields and were morphometrically analysed using Image ProPlus (Media Cybernetics Inc, Rockville, MD) performed in a blinded manner on coded slides. Eosinophil count in myocardial samples was determined by counting Luna^+^ cells in high power field and expressed as eosinophils per field using an optical microscope Leica DMD108 (Leica Microsystems, Wetzlar, Germany).

**References**

1. Bodi V, Sanchis J, Lopez-Lereu MP, Losada A, Núñez J, Pellicer M, et al. Usefulness of a comprehensive cardiovascular magnetic resonance imaging assessment for predicting recovery of left ventricular wall motion in the setting of myocardial stunning. J Am Coll Cardiol. 2005:46: 1747-1752.

2. Bodi V, Sanchis J, Núñez J, Mainar L, Lopez-Lereu MP, Monmeneu JV, et al. Prognostic value of a comprehensive cardiac magnetic resonance assessment soon after a first ST-segment elevation myocardial infarction. JACC Cardiovasc Imaging. 2009:2: 835-842.

3. Rios-Navarro C, Hueso L, Miñana G, Nuñez J, Ruiz-Sauri A, Sanz MJ, et al. Coronary serum obtained after myocardial infarction induces angiogenesis and microvascular obstruction repair. Role of hypoxia-inducible factor-1A. Rev Esp Cardiol. 2018;71: 440-449.

4. Hervas A, de Dios E, Forteza MJ, Miñana G, Nuñez J, Ruiz-Sauri A, et al. Intracoronary infusion of thioflavin-S to study microvascular obstruction in a model of myocardial infarction. Rev Esp Cardiol. 2015;68: 928-934.
